# Robust Automated Truncation Point Selection for Molecular Simulations

**DOI:** 10.1021/acs.jctc.4c01359

**Published:** 2024-12-23

**Authors:** Finlay Clark, Daniel J. Cole, Julien Michel

**Affiliations:** † EaStCHEM School of Chemistry, University of Edinburgh, David Brewster Road, Edinburgh EH9 3FJ, U.K.; ‡ School of Natural and Environmental Sciences, Newcastle University, Newcastle upon Tyne NE1 7RU, U.K.

## Abstract

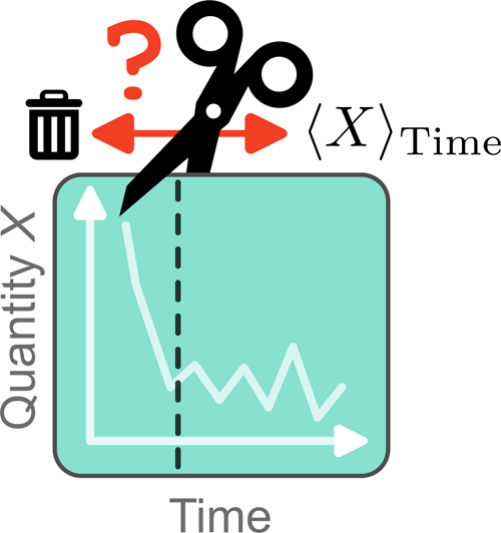

Quantities calculated from molecular simulations are often subject to an initial bias due to unrepresentative starting configurations. Initial data are usually discarded to reduce bias. Chodera’s method for automated truncation point selection [J. Chem. Theory Comput. 2016, 12, 4, 1799–1805] is popular but has not been thoroughly assessed. We reformulate White’s marginal standard error rule to provide a spectrum of truncation point selection heuristics that differ in their treatment of autocorrelation. These include a method effectively equivalent to Chodera’s. We test these methods on ensembles of synthetic time series modeled on free energy change estimates from long absolute binding free energy calculations. Methods that more thoroughly account for autocorrelation often show late and variable truncation times, while methods that less thoroughly account for autocorrelation often show early truncation, relative to the optimal truncation point. This increases variance and bias, respectively. We recommend a method that achieves robust performance across our test sets by balancing these two extremes. None of the methods reliably detected insufficient sampling. All heuristics tested are implemented in the open-source Python package RED (github.com/fjclark/red).

## 1 Introduction

Quantities calculated from molecular simulations are often subject to initial bias due to unrepresentative starting configurations. The resultant systematic error can be reduced by truncating data from the start of the simulation and calculating the quantity of interest using the remaining data. However, discarding too many data unnecessarily increases random error. It is common practice to select a fixed truncation point, which is unlikely to be optimal across many simulations, or to select by visually inspecting the data, which is only feasible for a few simulations. Robust automated truncation point selection methods are required to minimize errors in calculated quantities while facilitating automation. Here we address the selection of an optimal truncation point given some data, and not the much harder problem of detecting when sufficient data have been collected (so that enough representative configurations of the equilibrium distribution have been sampled). Therefore, we refer to “truncation point selection” and not “equilibration detection”, which might be expected to refer to the latter problem. We define the optimal truncation point to be that minimizing the root-mean-square error (RMSE) compared to the value which would be obtained with infinite sampling.

Two common heuristics for automated truncation point selection in molecular simulations are those of Yang et al.^1^ and Chodera.^2^ Yang et al. proposed a method based on reverse cumulative averaging, where a final “equilibrated” portion of the data are assumed to be Gaussian. The “equilibrated” region is extended in the reverse direction until significant deviation from this distribution is detected, indicating “unequilibrated” data. However, the assumption of normality is not always justified,^2,3^ and the robust selection of an initial “equilibrated” region may be challenging. Chodera suggested selecting the truncation point which maximizes the effective sample size, demonstrating the method on a simple system with rapid convergence and short correlation times. However, this method is prone to selecting late truncation points for more correlated data from more realistic applications.^4,5^

There is a substantial literature on truncation point selection outside the field of molecular simulation.^6−9^ In particular, the marginal standard error rule (MSER) family of methods has been found to be simple, effective, and easily automatable.^7,8,10^ These methods select the truncation point by minimizing the marginal standard error, and are very similar to Chodera’s method of maximizing the effective sample size; the main difference is that the MSER methods popular in operational research account for correlation less thoroughly, if at all, and their applicability to correlated molecular simulation data is uncertain.

Here, we compare the performance of a spectrum of MSER methods which apply increasingly rigorous techniques to account for correlation. These include the original MSER method and a method which is effectively identical to Chodera’s heuristic.^2,7^ To rigorously assess their performance, we test these methods on synthetic data modeled on long absolute binding free energy calculations for a variety of protein–ligand complexes. This work complements the recent study of Oliveira et al. by testing a wide range of MSER methods and quantitatively assessing their performance.^11^ We limit ourselves to the analysis of single runs, but note that truncation point selection and uncertainty quantification based on an ensemble of repeat runs are likely to be more robust, if more expensive.^4^ All methods discussed are implemented in the open-source Python package RED (robust equilibration detection, where equilibration is used in the sense of finding the optimal truncation point). This is available at github.com/fjclark/red and provides alternatives for the PyMBAR timeseries “detect_equilibration” function.^2,12^

## 2 Theory

### 2.1 Bias and Standard Deviation

As discussed by Chodera,^2^ we are usually interested in the ensemble average of a quantity *A*(***x***)
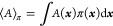
1where ***x*** is the vector of the system’s phase-space coordinates and π(***x***) is the probability of observing ***x*** in the ensemble of interest. ⟨*A*⟩_π_ is often estimated with ⟨*A*⟩_[*n*_0_,*N*]_, the average of a series of samples from a simulation
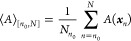
2where *N* is the total number of samples, *n*_0_ is the number of the first sample used in the average (earlier samples are discarded), *N*_*n*_0__ = *N* – *n*_0_ + 1, and ***x***_*n*_ is the vector of phase-space coordinates for the *n*th sample of the system. Assuming ergodicity, in the limit of an infinite number of samples, *N*_*n*_0__, ⟨*A*⟩_[*n*_0_,*N*]_ = ⟨*A*⟩_[*n*_0_,∞]_ = ⟨*A*⟩_π_.^13^ With finite sampling, ⟨*A*⟩_[*n*_0_,*N*]_ is an approximation of ⟨*A*⟩_π_ with an associated error. The expected root-mean-squared error (RMSE) of the estimate can be separated into contributions from bias and variance
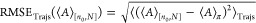
3
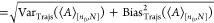
4
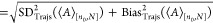
5where ⟨···⟩_Trajs_ indicates an average over the ensemble of all possible simulation trajectories with initial phase-space coordinates ***x***_**0**_ taken from some allowed set (often with the same positions). Var_Trajs_(⟨*A*⟩_[*n*_0_,*N*]_), Bias_Trajs_(⟨*A*⟩_[*n*_0_,*N*]_), and SD_Trajs_(⟨*A*⟩_[*n*_0_,*N*]_) denote the variance, bias, and standard deviation of ⟨*A*⟩_[*n*_0_,*N*]_ over the ensemble of trajectories. The (well-known) full derivation of eq 5 is given in Section S1.^2^ Explicitly, the expected bias and variance over the ensemble of trajectories are
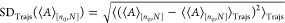
6
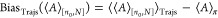
7These terms oppose each other—choosing an early truncation point (low *n*_0_) will produce a larger Bias_Trajs_ due to the inclusion of early unrepresentative samples, and a smaller SD_Trajs_ due to the inclusion of more samples (assuming similar starting configurations). Conversely, late truncation points reduce Bias_Trajs_ but increase SD_Trajs_. When truncating simulation data, the objective is to minimize RMSE_Trajs_ by balancing Bias_Trajs_ and SD_Trajs_.

### 2.2 Heuristics for Truncation Point Selection

We take “optimal” to mean “minimizing RMSE_Trajs_(⟨*A*⟩_[*n*_0_,*N*]_)”, and we aim to select the optimal truncation point. This is impossible in practice because RMSE_Trajs_(⟨*A*⟩_[*n*_0_,*N*]_) as a function of *n*_0_ is unknown. However, many heuristics have been proposed. White’s MSER selects an *n*_0_ by minimizing the marginal standard error of the mean of *A*,^7^ and is usually presented without explicitly accounting for autocorrelation
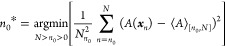
8where *n*_0_* is the selected *n*_0_. We present a more general formulation of MSER where autocorrelation may be explicitly accounted for
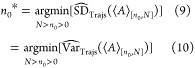
9where the hat operator indicates an estimate made from the available trajectory (or trajectories). Equations 9 and eq 10 are equivalent because the same truncation point minimizes the variance and the standard deviation. A naïve estimator of Var_Trajs_ is
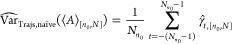
11
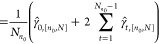
12where γ̂_*t*,[*n*_0_,*N*]_ is the estimate of the autocovariance at lag *t*. This measures the degree of linear dependence between values in a time series separated by *t* samples
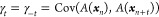
13
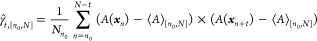
14Cov denotes covariance. We have assumed time-reversibility, hence γ_*t*_ = γ_–*t*_ and eq 11 simplifies to eq 12.

It is helpful to compare eq 12 to the familiar variance of the mean formula which assumes that samples are uncorrelated
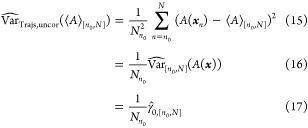
15This shows that the correlated variance estimate should always be larger than the uncorrelated estimate (when the correlations are positive). This is due to the autocovariance terms for lags greater than 0, which are missing from eq 17. When the uncorrelated variance estimate is used in the general MSER (eq 10), the traditional MSER (eq 8) is recovered. However, samples from molecular simulations are often strongly positively autocorrelated, meaning that eq 15 underestimates the true variance of the mean.

For simplicity, we do not correct for bias in our autocovariance estimates arising because the true mean is unknown.^14,15^ Additionally, we do not correct autocovariance terms for the finite size of the time series used to estimate them (we divide by *N*_*n*_0__ although there are only *N*_*n*_0__ – *t* terms in the sum). While this means that ⟨γ̂_*t*,[*n*_0_,*N*_*a*_]_⟩_Trajs._ ≠ ⟨γ̂_*t*,[*n*_0_,*N*_*b*_]_⟩_Trajs._ ≠ ⟨γ̂_*t*,[*n*_0_,∞]_⟩_Trajs._, where *N*_*a*_ ≠ *N*_*b*_, they will be very close to equal for the dominant autocovariance terms where *t* ≪ *N*_*n*_0__, and the lack of correction allows the variance of ⟨*A*⟩_[*n*_0_,*N*]_ to be estimated by summing the uncorrected γ̂_*t*,[*n*_0_,*N*]_ terms (Section 2.3).

 can be thought of as a surrogate for RMSE_Trajs_(⟨*A*⟩_[*n*_0_,*N*]_) which can be calculated with data from a single simulation; by picking the truncation point which minimizes , we hope to minimize RMSE_Trajs_(⟨*A*⟩_[*n*_0_,*N*]_). This is reasonable because at sufficiently late truncation points when the bias is negligible, often  becomes a good estimate of the Var_Trajs_(⟨*A*⟩_[*n*_0_,*N*]_) component of RMSE_Trajs_(⟨*A*⟩_[*n*_0_,*N*]_), which penalizes late truncation. For early truncation points, increasing bias increases , mimicking the Bias_Trajs_(⟨*A*⟩_[*n*_0_,*N*]_) contribution to RMSE_Trajs_(⟨*A*⟩_[*n*_0_,*N*]_) which favors late truncation. This relies on the violation of the time-reversibility assumption by the bias at early times to inflate , whereas Var_Trajs_(⟨*A*⟩_[*n*_0_,*N*]_) may not increase with increasing bias.

Chodera proposed choosing the truncation point which maximizes the effective sample size, which can be expressed as
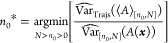
18which is very similar to the general MSER (eq 10). It differs only by the factor of . This will decrease sensitivity to bias compared to MSER, because an initial transient will increase , but this effect is expected to be small when autocorrelation is thoroughly accounted for (by considering all important terms in the autocorrelation function), as in Chodera’s work.^2^ Hence, the main difference between MSER and Chodera’s method lies in the calculation of . Therefore, we use eq 10 to select truncation points throughout this work, and compare different methods to calculate .

### 2.3 Calculation of the Variance of the Mean

All truncation point selection heuristics tested in this work are special cases of the general MSER (eq 10). They differ only in the way the variance of the mean is estimated. Calculating  by adding all autocovariance terms (eq 12) is problematic because at long lag times, these become dominated by noise. This makes  noisy.^16^ Instead, we consider a spectrum of methods which differ in the number and weighting of terms in the autocovariance sum they consider.

As discussed previously, the simplest method is simply to ignore all γ̂_*t*,[*n*_0_,*N*]_ terms when *t* > 0, as in the original MSER (eq 15). This will substantially underestimate the variance of the mean when *A* is highly correlated.

Lag window estimators of the variance of the mean take the form
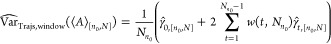
19where *w*(*t*, *N*_*n*_0__) is a weight function (“lag window”), which may depend on *N*_*n*_0__. *w*(*t*, *N*_*n*_0__) is greater than or equal to 0 and usually no larger than 1. This reduces the noise from later autocovariance terms by down-weighting them.^17^ Larger lag windows will generally underestimate the autocovariance less, at the expense of more noise.

A closely related method is batch means.^18^ The data are split into blocks of size *b*, and the variance of the overall mean is estimated from the variance of the block means
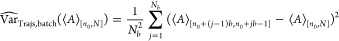
20where *N*_*b*_ = *N*_*n*_0__/*b* is the number of blocks, assuming *b* is a factor of *N*_*n*_0__. This relies on the fact that the batch means become uncorrelated as *b* → ∞. This method implicitly accounts for autocorrelation in the MSER-*m* family of methods, where the data are block-averaged using blocks of size *m* before MSER is applied.^10,19^ However, using overlapping batches, rather than the nonoverlapping batches in eq 20, yields a less noisy variance estimate.^20,21^ The overlapping batch means variance estimate is effectively equivalent to a lag window estimate (eq 19) using a Bartlett lag window (a kind of triangular window) of the appropriate size,^22^ and therefore eq 20 can be thought of as a noisy (if cheaper) special case of eq 19. Hence, we do not consider the batch means method. The circular bootstrap and moving blocks bootstrap methods are also asymptotically equivalent to the overlapping batch means method.^23−26^

A final class of methods terminates the sum of autocovariance terms according to Markov chain-specific criteria. These are Geyer’s initial sequence estimators.^16^ Geyer noted that for stationary, reversible, and irreducible Markov chains, the sequence of sums of pairs of covariance terms
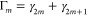
21is positive, decreasing, and convex. Geyer’s initial positive, monotone, and convex sequence estimators impose each of these properties (and all preceding properties), respectively. For example, the initial positive sequence (IPS) estimator of the variance of the mean is
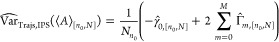
22
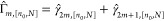
23where *M* is the largest value of *m* for which Γ̂_*m*,[*n*_0_,*N*]_ > 0, *m* ≤ *M*. Chodera used a similar method where the autocovariance sum is truncated at the first negative autocovariance term.^2,12^ Of the methods discussed, these estimators tend to produce the largest estimates of autocorrelation and hence the largest and most realistic estimates of the variance of the mean. While many other schemes exist to estimate the autocovariance function and variance of the mean,^23,27−36^ a detailed comparison is beyond the scope of this study.

Finally, we note that all single-run estimates of the variance of the mean are likely to be severe underestimations, due to the tendency of molecular simulation trajectories to become trapped in local minima.^37^

### 2.4 Assessing Truncation Point Selection Algorithms

A variety of metrics have been used to assess truncation point selection algorithms in the operations research literature, including percentage bias removed by truncation, the closeness of the truncation point to the first unbiased point, the coverage of the true mean by the calculated confidence intervals, and the variability of the truncation point.^8,19,38^ However, we believe that the most natural metric is the RMSE of the estimated means to the true mean over an ensemble of test trajectories.^2^ This most closely reflects our intentions when using these heuristics—to minimize error to the true value.

For these RMSEs to be meaningful, we need to test with ensembles of trajectories which reflect real use cases, and we must know, or have good estimates of, ⟨*A*⟩_π_ for observables of interest. This is not straightforward with real simulation data; systems complex enough to be interesting often require long simulations to remove bias and accurately estimate ⟨*A*⟩_π_, and are often expensive to simulate, making the generation of an ensemble of trajectories unfeasible. Cheaper trajectory ensembles can be generated using simpler test systems, such as Chodera’s liquid argon,^2^ but this does not reflect real use-cases such as protein–ligand binding free energy calculations.^39^ Simple models used in the operations research literature have the same problem here.^8,19,38^ We attempt to address this issue by fitting models to data from compute-intensive absolute binding free energy calculations. From these models, we can cheaply generate ensembles of synthetic trajectories with exactly known properties.

## 3 Methods

We began with data from absolute binding free energy calculations. We used long calculations to allow more time for the initial transient to decay and to reduce errors in the estimated autocorrelation functions. To make the data more representative of absolute binding free energy calculations in general, we used data from a diverse range of protein–ligand complexes. The real data are computationally expensive to generate and unsuitable for testing since the infinite-sampling results are unknown. Therefore, the initial transients and autocorrelation functions of the real data were estimated and used to generate large numbers of correlated synthetic time series from uncorrelated Gaussian noise. The properties of the synthetic time series did not need to exactly match the real data they were modeled on, only to be reasonably representative of absolute binding free energy data. Because the infinite sampling time results were known for the synthetic time series, truncation point selection heuristics could be evaluated by their RMSE to the infinite sampling time results.

### 3.1 Generation of Simulation Data

Data were taken from our recent absolute binding free energy study—specifically, for the long (30 ns per simulation) nonadaptive runs.^4^ Simulation details are given in Clark et al.^4^ In free energy calculations, the Hamiltonian, *H*, is parametrized by λ. λ scales the strength of intermolecular interactions of the ligand in absolute binding free energy calculations. The real data consist of simulations run at λ = 0, where (a subset of) interactions are fully active, at λ = 1, where these interactions are removed, and at several intermediate values.^4^ An important quantity is the gradient , as the free energy change for a transformation can be estimated by integrating the mean gradient over λ. Therefore, we base our testing on the time series of (integrated) . All sampled  are provided on Zenodo.^4,40^ To provide challenging test data, we mainly use data from the “bound vanish” stage of the thermodynamic cycle, where the ligand intermolecular Lennard-Jones terms are progressively removed while the ligand is restrained in the protein binding site. The bound vanish stage generally shows the most pronounced initial transient and longest correlation times. The data include 5 different test systems (see Section S3 of Clark et al. for more details) with ligands ranging from small and rigid benzene up to a 70-atom ligand, ensuring diverse initial transient and autocorrelation behavior.^4^ The complexes were the L99A mutant of T4 lysozyme (T4L) with benzene,^41,42^ human macrophage migration inihibition factor (MIF) with the ligand MIF180,^43,44^ mouse double minute 2 homolog (with a truncated lid) with the ligands Pip2 and Nutlin,^45^ and phosphodiesterase 2a with the ligand “P10”.^46^ Some analysis was also performed for the free vanish stage, where the ligand intermolecular Lennard–Jones interactions are removed in water. All simulations were performed with a 4 fs time step and  was saved every 200 steps. For each system, a time series of estimated free energy change against simulation time was produced by integrating  over λ using the trapezoidal rule.^47^ Hence, the *A* of previous sections is replaced with Δ*G*. These time series were averaged over the 5 replicate runs performed for each system to provide less noisy estimates of model parameters discussed in Section 3.2.

### 3.2 Generation of Synthetic Data

**Figure 1:**
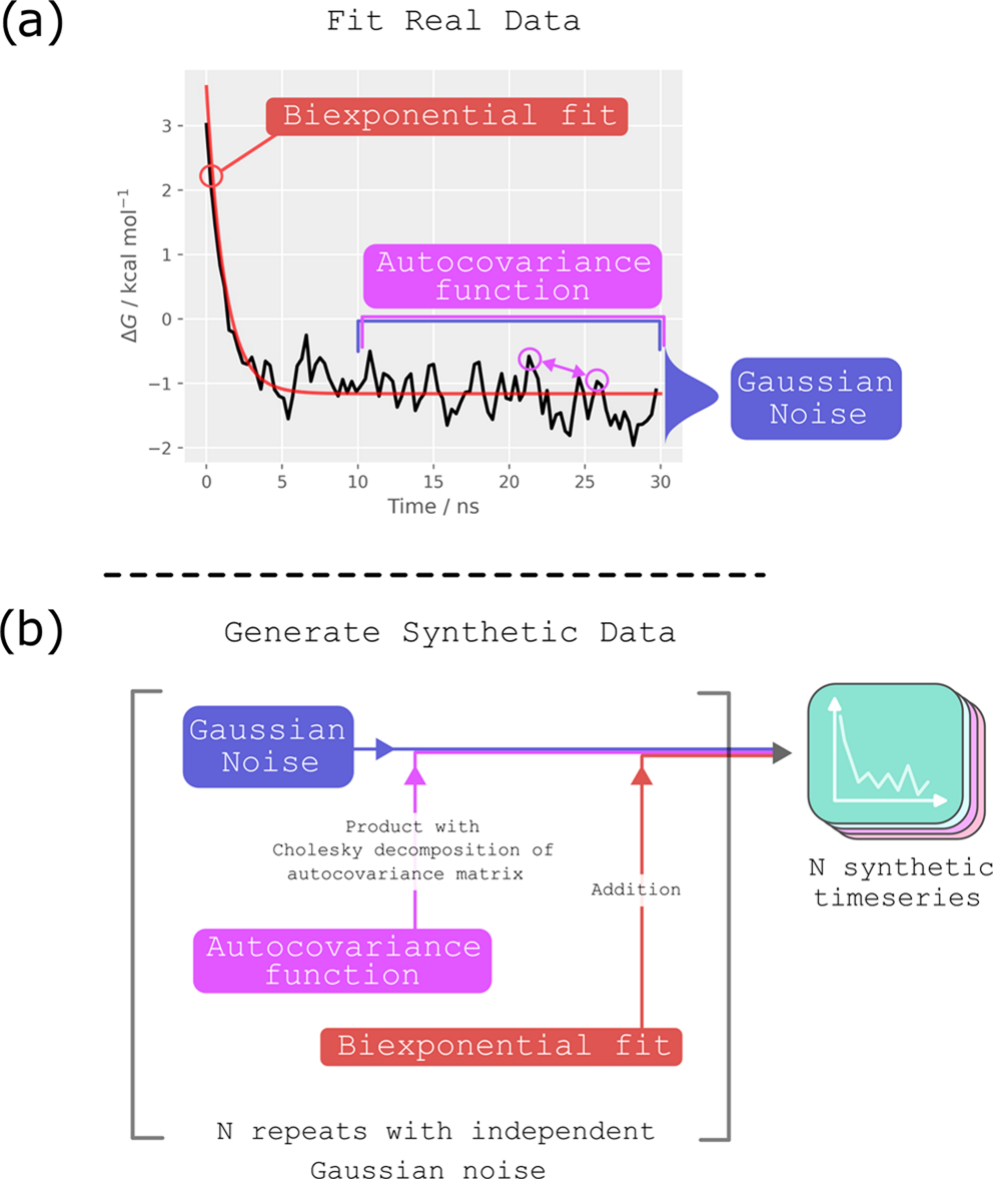
Creation of synthetic data sets modeled on real data. (a) A 30 ns time series of free energy change estimaters against simulation time was obtained from an absolute binding free energy calculation (here we averaged over 5 repeats for the Lennard–Jones term removal stage when the ligand is bound to the protein). The initial transient was captured with a biexponential fit. The final 20 ns generally showed no substantial overall trend and were approximately Gaussian. These data were used to fit an autocovariance function. (b) To generate synthetic time series, we started with a vector of uncorrelated Gaussian noise. The desired autocorrelation structure was reintroduced by taking its product with the Cholesky decomposition of the autocovariance matrix, and the initial transient was reintroduced by adding the biexponential fit. This was repeated *N* times with different Gaussian noise (but the same autocovariance function and biexponential trend) to generate *N* synthetic time series. We used *N* = 1000.

The general strategy for modeling the data is illustrated in Figure 1: initial transients were fitted to the first 10 ns of simulation data, while autocovariance functions were fit to the final 20 ns of approximately stationary data. These were used to produce large ensembles of synthetic time series, where each synthetic time series was generated from different uncorrelated Gaussian noise, but shared the same initial transient and autocovariance functions.

In detail, the bound vanish stage time series generally showed no substantial drift over the final 20 ns (from 10 to 30 ns, Figures S1 and S2). Therefore, the “true” infinite sampling time free energy changes were calculated as the mean over the last 20 ns, and were subtracted from the time series. The initial transients were generally well-fit by a single exponential decay (Figure S3), although some quickly decaying bias was evident for most systems at short time scales. To account for this, a second exponential with short half-life was fit after subtracting the first exponential, and retained only if the pre-exponential term was positive (Figure S4).

Autocovariance functions were calculated for each system using the final 20 ns portions of the time series. The first two points were calculated directly from the averaged time series, while the remaining points were obtained by interpolating the convex Γ function computed following Geyer (Figures S6 and S7).^16^Figure S5 shows that the final 20 ns portions of the time series were well-modeled by Gaussian distributions (although some systems showed small but significant deviations from normality). Therefore, synthetic ensembles of trajectories were generated for each system starting from vectors of uncorrelated standard normal noise. 1000 noise vectors were generated for each system. The Cholesky decompositions of the autocovariance matrices were obtained and the resulting lower-triangular matrices were used to reintroduce the desired correlation structure to the uncorrelated noise vectors. The exponentials were then added to introduce the initial transients. Examples of synthetic time series are shown in Figures S9 and S10.

Synthetic ensembles of trajectories were also created for the free vanish stage for the T4L and PDE2a systems, which were chosen to represent highly uncorrelated and relatively correlated time series, respectively (Section S3). Trajectory ensembles were generated as for the bound vanish stage, except no exponential trends were fit.

The synthetic data will not be perfect models of the true data, because the initial transients, autocovariance functions, and infinite sampling time “true” free energy changes will not be perfectly estimated. This does not matter, so long as the properties of the synthetic time series are reasonably representative of absolute binding free energy data in general. Hence, we are not concerned with precisely modeling specific features of the real data, for example, the large drop observed for MDM2-Nutlin Repeat 1 around 11 ns (Figure S2). Relatedly, the mean time series for T4L drifts from ≈3.5 to 4.0 kcal mol^–1^ between 10 and 30 ns, in the opposite direction to the apparent initial transient. This appeared to violate our assumption of no drift in this region and produced a long-tailed autocovariance function (Figure S6 and Table 1). However, it does not matter if this does not match the “true”, infinite sampling autocovariance function for T4L, because the autocovariance function appears realistic:^30,48^ it shows a fast initial decay followed by long, approximately exponential tail (Figure S8).

**Table 1:** Model Parameters Fitted to Bound Vanish Stages of Absolute Binding Free Energy Calculations^a^

	half-life (ns)	*a* (kcal mol^–1^)	fast half-life (ns)	fast *a* (kcal mol^–1^)	total variance (kcal^2^ mol^–2^)	max lag index
T4L	0.33	0.86	∞	0	110	7175
MIF	0.88	4.7	0.0040	14	55	749
MDM2-Nutlin	1.6	2.6	0.0052	21	81	577
MDM2-Pip2	0.80	3.6	0.0057	12	41	335
PDE2A	0.33	13	0.019	13	520	1699

a Total variance refers to the total variance of the mean, obtained by summing the autocovariance series from −max lag index to + max lag index, where the series and maximum lag indices were estimated according to Geyer’s initial convex sequence rules. “*a*” refers to the pre-exponential factors.

### 3.3 Testing of Heuristics

Three types of method were tested for calculating the variance: an uncorrelated estimate which used eq 17 (“Uncorrelated Estimate”, equivalent to White’s original MSER), window methods using eq 19 with window sizes 5, 50, and , and initial sequence methods based on Geyer’s initial positive, monotone, and convex sequence rules.^16^ We tested the  window size due to its use in selecting batch and window sizes for variance estimation.^18,49^ We use triangular windows of the form
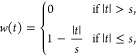
24where *s* is the window size. Chodera’s method for terminating the autocovariance function is not one of Geyer’s initial sequence methods. However, we included it with the initial sequence rules (as “Initial Sequence: Chodera”) due to its similarity. We also tested a variant of Geyer’s initial convex sequence method where each successive variance calculation (with increasing truncation) was restricted to a Γ series (eq 23) no longer than that from the previous truncation point (named “Initial Sequence: Smoothed Lag Convex”). In all cases, we did not calculate the marginal standard error for the last 10% of the data, to avoid noisy estimates producing very late truncation.^50^

Each method was applied to every member of the synthetic ensembles after truncating the synthetic data after 8 ns, which is fairly typical of an absolute binding free energy calculation.^43^ In addition to the “Standard” data sets (8 ns truncation and generated as described above), we tested all methods on a “Short” data set where the truncation point was 0.2 ns, a “Subsampled” data set where 99 of every 100 data points were discarded, a “Noisy” data set where the autocovariance terms were scaled up by √5, and a “Block-Averaged” data set, where successive blocks of 100 data points were replaced by their averages. Reported uncertainties in RMSE_Trajs_(⟨Δ*G*⟩_[*n*_0_,*N*]_), SD_Trajs_(⟨Δ*G*⟩_[*n*_0_,*N*]_), and SD_Trajs_(⟨Δ*G*⟩_[*n*_0_,*N*]_) for each of the heuristics are 95% confidence intervals which were calculated by bootstrapping synthetic trajectories 10 000 times with replacement.

All tested methods were implemented in the open-source Python package RED (Robust Equilibration Detection, where “equilibration” is used in the sense of finding the optimal truncation point) available at github.com/fjclark/red. A complete workflow to reproduce the study beginning from the absolute binding free energy gradient data is provided at github.com/michellab/Robust-Equilibration-Detection-Paper and all data are provided on Zenodo.^51^

## 4 Results

### 4.1 Initial Sequence Methods are Prone to Over-Discarding

To understand how the methods performed on time series with no initial transient, we applied them to the free vanish stage synthetic trajectory ensembles. We used the T4L system (benzene in water) and the PDE2a system (ligand P10 from Huggins in water) as examples of relatively low variance and correlation, and relatively high variance and correlation systems, respectively.^46^ Details of the fitting procedure and parameters are given in Section S3. As the synthetic data contained no biases, the optimum truncation time was 0 ns and late truncation indicated problems with the heuristics.

**Figure 2:**
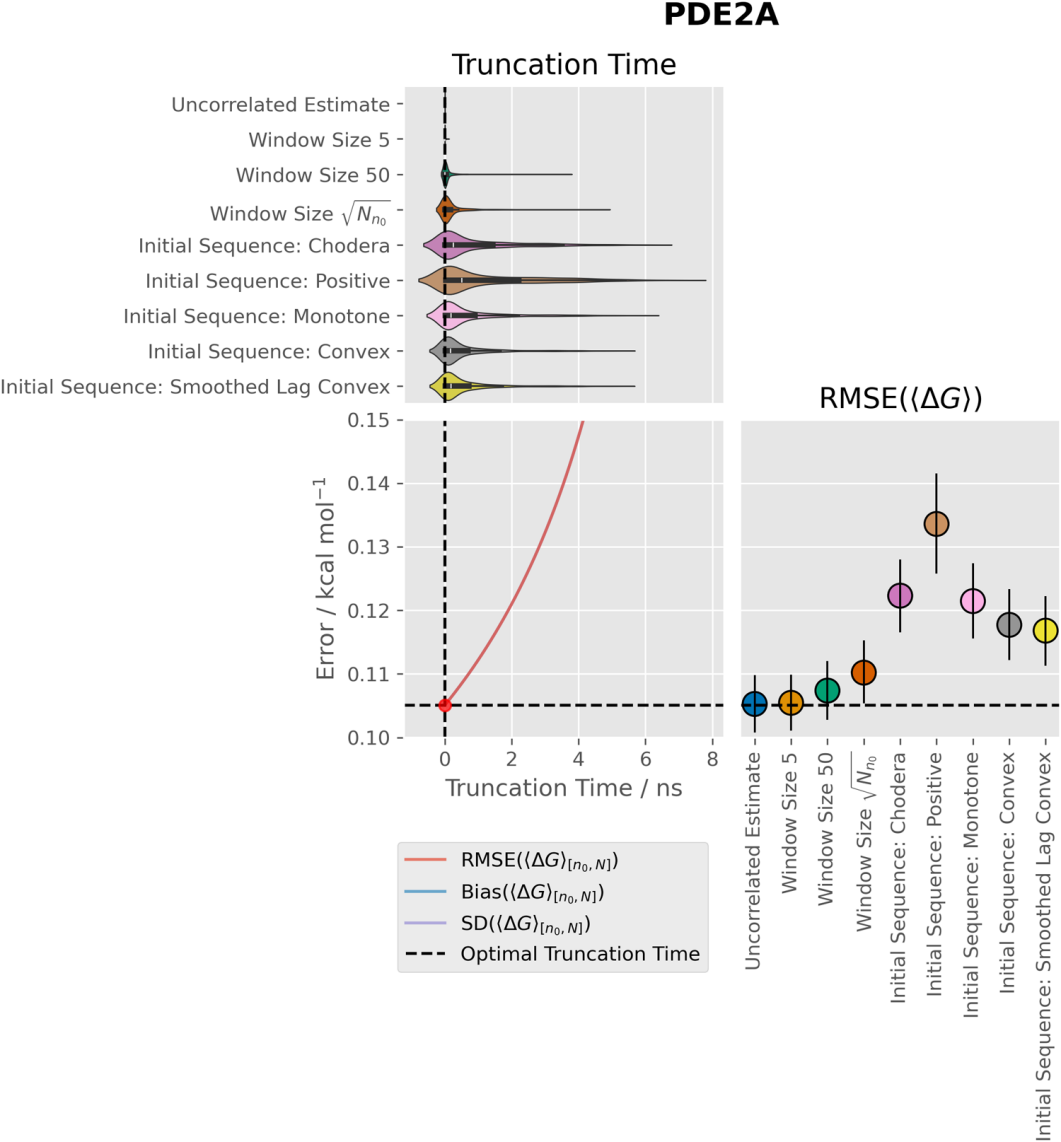
Discard times, RMSEs, and underlying time series properties for the free vanish stage for PDE2A. The top panel shows kernel density estimates of the distributions of times discarded with each method. The bottom left panel shows the RMSEs which would be obtained with an infinitely large ensemble of synthetic time series with fixed truncation points. The red dot indicates the optimum fixed-time truncation point. This is at time 0 ns as there is no bias. The bottom right panel shows the RMSEs obtained with each generalized MSER method. Uncertainties are 95% confidence intervals obtained with 10 000 iterations of bootstrapping with replacement. Note that the y-axis is shifted from 0 to make the differences between methods clearer.

For the relatively low variance and correlation T4L system, all methods consistently selected discard times very close to 0 ns, producing RMSEs within uncertainty of the optimal fixed truncation time limit (at 0 ns—Section S4). It was reassuring to observe this expected behavior. However, for the relatively high variance and correlation PDE2a system, methods which more fully accounted for autocorrelation were increasingly prone to overdiscarding data (Figure 2). This was particularly true for the initial sequence methods, which occasionally discarded over half of the data despite the lack of any bias. This was reflected by increases in the RMSEs. The “Initial Sequence: Positive” method should produce the largest variance of the mean estimates for a given time series because it chooses the latest truncation point for the autocovariance series and does not reduce the autocovariance sum by enforcing monotonicity or convexity of the Γ series. This method was the most prone to overdiscarding data, corroborating the trend that generalized MSER methods which produce larger variance of the mean estimates are more prone to erroneously discarding data. Within the initial sequence methods, applying Geyer’s initial monotone and convex rules slightly reduced erroneous late truncation compared to the initial positive sequence method.

Using the first 100 PDE2a synthetic trajectories, we verified that the maximum effective sample size heuristic implemented in PyMBAR’s timeseries module gave identical results to the implementation in RED.^2,12^ We then compared the truncation points selected using this method and the equivalent minimum marginal standard error heuristic (see eqs 18 and 10), where we did not employ the adaptive integration scheme described by Chodera et al.^12^ As expected, the truncation points selected were generally very similar: identical truncation times were selected in 81% of cases and the difference was less than 0.1% (of the total time series length) for 95% of the trajectories. The minimum standard error method showed a slight bias for later truncation times over the maximum effective sample size method—the mean (ESS—MSE) difference was −0.34% of the total time series length. However, we found that using the marginal standard error method fixed several problem cases where time series (not the synthetic data discussed above) were contaminated by one or a few very different initial samples, which the effective sample size method failed to remove.

We also examined the effect of the adaptive integration scheme described by Chodera et al. on the same trajectories.^12^ This scheme reduces the computational cost by calculating the autocorrelation less frequently at greater lag times, but we found that it produced more erroneously late truncation times (Section S5).

###  Window Method Balances Bias Sensitivity and Variability

4.2

Next, we tested the methods on the “Standard” synthetic ensembles generated from the bound vanish stages. The fitted parameters used to generate the synthetic ensembles were fairly diverse, with the variance of the mean estimates, max lag index (for the initial convex sequence estimate of the autocovariance series), and pre-exponential factors for the “slow” exponential fits varying over an order of magnitude (Table 1). The half-lives of the “slow” exponential fits varied from 0.3 to 1.6 ns. Further details of the parameter fitting and synthetic data generation are given in Section S2.

**Figure 3:**
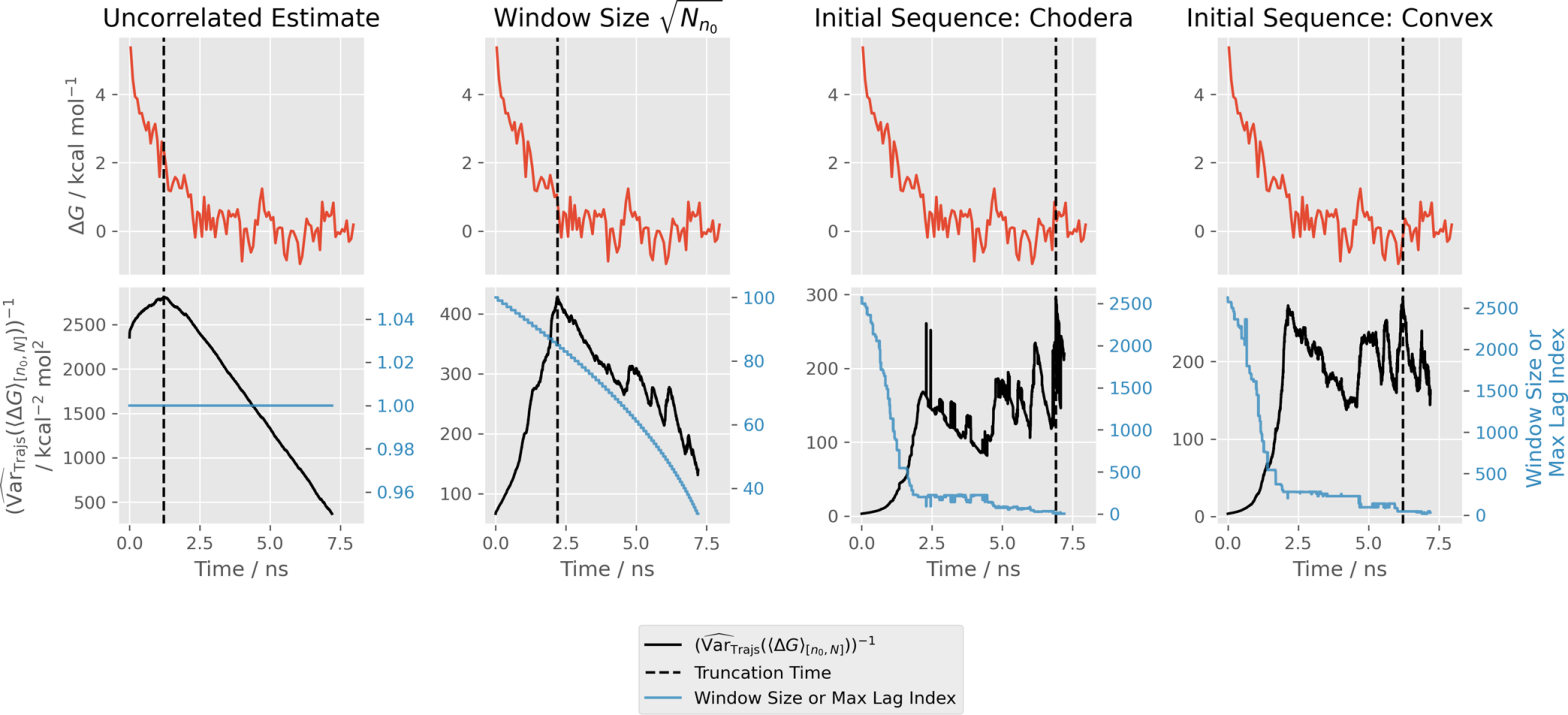
Performance of several generalized MSER heuristics on a single bound vanish synthetic times series for for MIF. To show features close to the selected truncation points, we show the inverse of the squared (marginal) standard error (). With the Chodera method, sudden dips in the maximum lag index cause spikes in the inverse  (two spikes are marked by the red asterisk). The time series have been block-averaged with 100 blocks to make the trends clearer.

We compared the ensemble RMSEs obtained by each generalized MSER method to the minimum possible RMSE obtained by applying the optimal fixed-time truncation to all time series. In general, methods which produced larger variance of the mean estimates were prone to choosing late truncation points, increasing SD_Trajs_(⟨Δ*G*⟩_[*n*_0_,*N*]_), while methods which underestimated the variance of the mean were prone to choosing early truncation points, increasing Bias_Trajs_(⟨Δ*G*⟩_[*n*_0_,*N*]_). This is illustrated for one of the synthetic MIF time series in Figure 3 (we show  in place of  to make the behavior in the region of minimum  clearer). The “Uncorrelated Estimate” (original MSER) is only sensitive to offset of the time series mean through the first term of the autocovariance function, γ̂_0,[*n*_0_,*N*]_ (eq 17). As a result, it produces underestimates of  which vary smoothly with increasing truncation but are insensitive to bias. This results in early truncation. The estimates of γ̂_0,[*n*_0_,*N*]_ quickly stabilize with increasing truncation and the variance of the mean estimates become proportional to . In contrast, the initial sequence methods are sensitive to offset of the time series mean through many more terms of the autocovariance function. As a result, offsetting the mean more effectively counterbalances the  term to increase the variance of the mean estimate. This prevents early truncation. However, the  estimates become much noisier, especially at late truncation times, which can produce spurious minima in the variance of the mean estimation, leading to late truncation. Sudden dips in  were often caused by sudden decreases in the maximum lag index used to calculate the autocovariance sum. For the Chodera method in particular, troughs at single values of *n*_0_ were often observed (Figure 3). However, issues with troughs in the maximum lag index were generally removed or reduced with increasingly stringent initial sequence methods. In general, Geyer’s initial monotone and convex sequence methods produced the smoothest traces of  of the “initial sequence” methods (including Chodera’s). The window method with window size  compromises the extremes of the uncorrelated estimate and the initial sequence methods by including enough treatment of correlation to avoid excessively early truncation, while avoiding noisy variance of the mean estimates which can produce late truncation.

**Figure 4:**
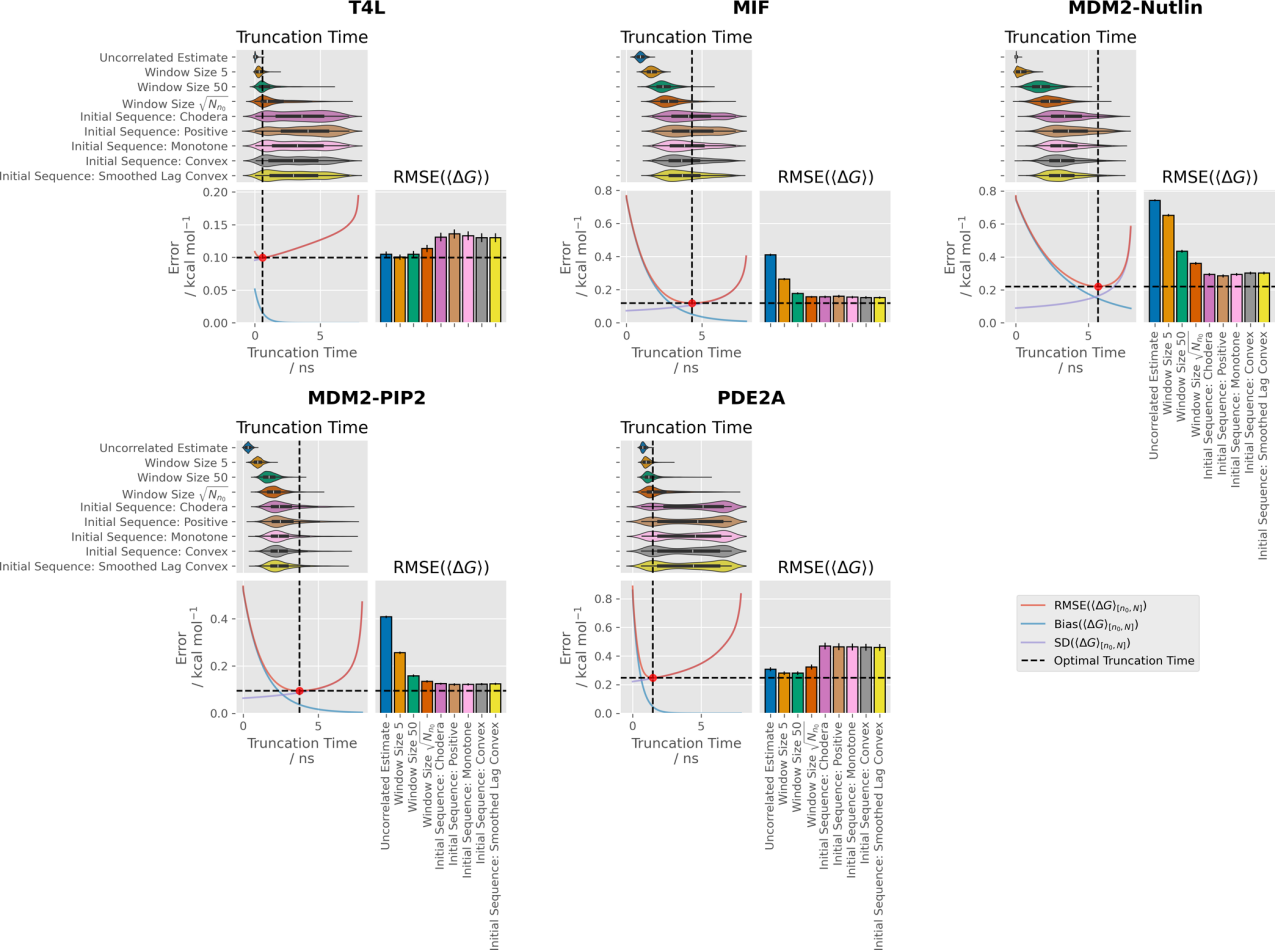
Discard times, RMSEs, and underlying time series properties for the bound vanish stage time series. Within each system block, the top panels show kernel density estimates of the distributions of times discarded with each method. The bottom left panels show the RMSEs which would be obtained with an infinitely large ensemble of synthetic time series with fixed truncation points. The red dot indicates the optimum fixed-time truncation point. Bottom right panels show the RMSEs obtained over the full synthetic ensemble for each method. Uncertainties are 95% confidence intervals obtained with 10 000 iterations of bootstrapping with replacement.

More quantitatively, the performance of all methods across the “standard” synthetic ensembles is compared in Figure 4 and Table 2. The test systems can be divided into two groups: those where the optimal fixed-time truncation point is around or above 50% of the simulation time (MIF, MDM2-Nutlin, and MDM2-Pip2), and those where it is substantially less (T4L, PDE2a). Unsurprisingly, methods prone to late truncation (the initial sequence methods), performed best in the first set, while methods prone to early truncation performed best in the latter set. For example, MDM2-Pip2 had a relatively late optimal fixed truncation time (around 4 ns). The uncorrelated estimate method generally truncated at less than 1 ns for this system, producing an ensemble RMSE around 4 times greater than the fixed truncation point minimum. For the window estimators, the mean truncation times increased and the ensemble RMSEs decreased with increasing window size up to the  window estimator—this produced an ensemble RMSD close to the optimal fixed truncation time limit and to the initial sequence estimator methods, which performed the best. In contrast, the PDE2a system had a relatively early optimal fixed truncation time (around 1.5 ns). Here, the best-performing methods were the window estimators with window sizes of 5 and 50, both with an ensemble RMSE of 0.28_0.27_^0.29^ kcal mol^–1^. The RMSE for the uncorrelated estimate and window size  methods were marginally worse (0.31_0.30_^0.32^ and 0.32_0.31_^0.34^ kcal mol^–1^, respectively), while the RMSEs for the initial sequence methods were substantially higher (≈0.46 kcal mol^–1^) due to late truncation. Worryingly, the initial sequence methods showed strongly bimodal distributions of discard times, centered around 1.5 and 7 ns. Hence, the strong bias sensitivity of the initial sequence methods comes with an increased propensity to truncate all data but the final local trough or plateau.

**Table 2:** Ensemble RMSEs for All Generalized MSER Heuristics for the “Standard” Bound Vanish Data^a^

method	T4L	MIF	MDM2-Nutlin	MDM2-Pip2	PDE2A
uncorrelated estimate	0.105_0.101_^0.109^	0.411_0.404_^0.417^	0.743_0.737_^0.749^	0.408_0.403_^0.413^	0.308_0.295_^0.321^
window size 5	0.100_0.096_^0.105^	0.264_0.257_^0.270^	0.653_0.646_^0.660^	0.256_0.251_^0.262^	0.280_0.268_^0.292^
window size 50	0.105_0.100_^0.110^	0.178_0.172_^0.184^	0.434_0.425_^0.443^	0.159_0.154_^0.164^	0.280_0.267_^0.293^
window size	0.114_0.108_^0.119^	0.157_0.151_^0.163^	0.361_0.352_^0.369^	0.135_0.130_^0.140^	0.323_0.306_^0.341^
initial sequence: Chodera	0.131_0.125_^0.138^	0.157_0.149_^0.164^	0.293_0.285_^0.302^	0.125_0.121_^0.130^	0.469_0.445_^0.493^
initial sequence: positive	0.136_0.129_^0.143^	0.161_0.153_^0.169^	0.285_0.276_^0.294^	0.122_0.117_^0.127^	0.465_0.440_^0.489^
initial sequence: monotone	0.133_0.126_^0.140^	0.155_0.148_^0.163^	0.294_0.285_^0.303^	0.122_0.118_^0.127^	0.463_0.439_^0.488^
initial sequence: convex	0.130_0.123_^0.137^	0.153_0.146_^0.160^	0.302_0.293_^0.311^	0.124_0.119_^0.128^	0.461_0.437_^0.485^
initial sequence: smoothed lag convex	0.130_0.124_^0.137^	0.152_0.145_^0.159^	0.302_0.293_^0.310^	0.125_0.120_^0.129^	0.459_0.435_^0.483^

a All values in kcal mol^–1^. Uncertainties are 95% confidence intervals obtained by bootstrapping over 10 000 iterations with replacement.

We found that the  window method was the most robust truncation selection heuristic on these data. Its intermediate treatment of autocorrelation balanced the propensities for early and late truncation shown by methods ignoring correlation, and methods more completely accounting for correlation, respectively. Methods at the extremes of the correlation spectrum performed well on the early truncation point set and poorly on the late truncation point set or vice versa. In contrast, the  window method never performed more than 29% worse (by RMSE) than the top-performing automated method. It also did not produce the wide and sometimes bimodal distributions of truncation times seen for the initial sequence estimators, which we view as an advantage in itself.

There were systems where all methods systematically underestimated the truncation point (particularly MDM2-Nutlin and MDM2-Pip2). However, the best-performing methods generally showed an ensemble RMSE close to the optimal fixed truncation point minimum, and the  window method never performed much worse. Figure 4 shows that the  window method would never perform much worse, and would often perform substantially better compared to a common default fixed truncation time of 20% (1.6 ns). Further analyses of the “standard” ensembles are presented in Section S6.

The left-most local minimum (LLM) method has been proposed to avoid the overtruncation of data with MSER.^9^ The left-most local minimum is selected as the truncation point, rather than the usual global minimum. However, we found that this produced very early truncation points when applied to the initial sequence generalized MSER methods, because the curves of MSE against truncation point were noisy and had early minima. For our data, it would be unhelpful to apply this to the other truncation heuristics, because they already showed a tendency toward early truncation.

The observation that sudden troughs in the maximum lag time led to instabilities with the Chodera method prompted us to try the “Initial Sequence: Smoothed Lag Convex” method, in which the maximum lag times at subsequent truncation times were never allowed to increase. Geyer’s initial positive, monotone, and convex sequence rules were also applied. However, the performance was very similar to the “Initial Sequence: Convex” method.

Oliveira et al. qualitatively tested MSER (original, batch, and LLM variants) in grand cannonical Monte Carlo simulations and concluded that it out-performed methods including Chodera’s maximum effective sample size heuristic, which they found prone to late truncation.^11^ However, we found that the original MSER method (“uncorrelated estimate”) often severely underestimated the optimal truncation time, producing very large RMSEs (for example, see MDM2-Pip2 in Table 2). A key difference between Oliveira et al.’s data and ours is that the initial transient is fairly hidden by the high variance of our data (see Figure S1 for the non block-averaged time series). It is likely important to account for autocorrelation to effectively recover the trend from the noise. This is not the case for Oliveira et al.’s data, where reasonable truncation times appear to be estimated without accounting for autocorrelation, avoiding the associated risk of late truncation. Hence, the optimal truncation point selection heuristic will likely depend on the properties of the data. However, as discussed below, the  window method remains a good choice for several variants of our synthetic data set.

### 4.3 Similar Results are Obtained with Noisier Data

To increase confidence that our results were generalizable, and to better understand the methods, we retested all heuristics on modified synthetic ensembles. Detailed results are given in Section S6.

“Noisy” ensembles were created in the same way as the “standard” data, but all autocovariance terms were increased by a factor of √5. This was intended to reverse the effect of averaging over the 5 repeat runs before the synthetic data parameters were extracted. The effect of the added noise was to shorten the optimum truncation time (Figure S17), which was reflected by earlier truncation time distributions for all heuristics. However, the relative performance of all methods on all systems remained very similar to the standard data (Figure S17 and Table S3).

To investigate whether these heuristics were applicable to simulations run at a single value of λ, rather than the free energy change integrated over λ, we modeled synthetic data on a a single λ state simulation. Data were modeled on a particularly noisy bound vanish λ state, which generally produced higher ratios of total variance to the slow exponential prefactor. However, the comparative performances of the heuristics were similar to the “standard” synthetic ensemble (Section S6).

### 4.4 Subsampling and Block Averaging Reduce Differences between the Methods Due to Reduced Autocorrelation

To check whether our results were affected by the frequency of data collection, we subsampled 1 out of every 100 data points. Reducing the number of data points increased the ratio of SD_Trajs_(⟨Δ*G*⟩_[*n*_0_,*N*]_) to Bias_Trajs_(⟨Δ*G*⟩_[*n*_0_,*N*]_), shifting the optimal truncation points to slightly earlier times (Figure S17). As the subsampling interval was a large fraction of the length of the autocorrelation functions used to generate the data (Table 1), this also dramatically reduced the autocorrelation of the time series. It was therefore unsurprising that all generalized MSER methods, which differ only in their treatment of autocorrelation, performed similarly on all subsampled trajectory ensembles (Table S4). The truncation time distributions for all methods became similar: those from the “uncorrelated estimate” generally shifted later, and the truncation time distributions from the initial sequence methods became narrower and shifted earlier. Unlike with the “standard” data, no methods produced RMSEs dramatically greater than the fixed truncation time minimum. For these reasons, it may seem tempting to subsample all time series before applying the heuristics. However, this is problematic—if the mean is calculated from the truncated subsampled data, it will have an substantially higher RMSE than for the original data, due to the loss of information in the discarded samples. Applying the truncation time from the subsampled data is also ill-advised because the subsampled data have earlier optimal truncation times than original data; the truncation times for most methods will be biased toward erroneously early times.

Block averaging is another method which reduces autocorrelation between consecutive data points. However, unlike subsampling, it does not affect the underlying bias-standard deviation trade-off of the time series, and the optimal fixed-time truncation points remain the same. As discussed in Section 2.3, estimation of the variance of the mean by block averaging is closely related to estimating the variance of the mean using window estimators with a Bartlett window.^20^ Hence, block averaging the data before analysis effectively turns the “uncorrelated estimate” into a window estimator, and increases the effective window sizes of the window size estimators. However, we repeated the tests after block averaging all “standard” time series (block size 100) as a way of simulating less correlated and less noisy data. Block averaging dramatically improved the performance of the “uncorrelated estimate” on the late truncation point systems (Figure S17 and Table S5), which was unsurprising given that this effectively became a window method with a large window. Generally, the distributions of discard times became wider and shifted to later times for all methods. For the early truncation point systems, this generally worsened the performance of the window estimators so that they became comparable with the initial sequence estimators. In terms of RMSE, the initial sequence methods usually did not perform significantly worse than without block-averaging. The bimodal discard time distributions observed for the initial sequence methods on the “standard” data set were observed for the window estimators for some systems.

### 4.5 All Methods Usually Fail to Detect Insufficient Sampling

This work compares heuristics for truncation point selection once sampling is complete. However, it would be useful if these methods could also detect when all samples are highly biased, indicating that few representative samples from the equilibrium distribution have been obtained and more sampling is required. In other words, it would be useful if truncation point selection heuristics could be used for equilibration detection. In the context of MSER, truncation times over 50% of the simulation time have been taken to indicate a lack of equilibration.^50^ We tested this heuristic by applying all generalized MSER methods to the first 0.2 ns of the “standard” synthetic data sets (Figure S17 and Table S6). For all time series, the optimal fixed-time truncation points were at over 50% of the total time, and usually close to 100%. PDE2a had large and quickly decaying bias at these short time scales (Table 1), which produced a steep decrease of RMSE with increasing truncation time compared to all other systems. For all other systems, the distributions of discard times were relatively narrow and centered at less than 0.05 ns for all methods. Hence, all methods failed to detect insufficient sampling according to the 50% time rule. However, for PDE2a, the size 50 window method and all methods with fuller treatment of correlation showed median discard times at greater than 50% of the total time. This indicates that when large and rapidly decaying initial transients dominate slowly increasing random errors, the 50% time rule may be effective as a simulation-stopping criterion (especially when used with generalized MSER methods which more fully account for autocorrelation). This appears to be the case for Oliveira et al.’s data. However, our results suggest that this is unlikely to be the case for most free energy calculations.

### 4.6 Practical Use of Generalized MSER Methods

#### 4.6.1 General Applicability

We have attempted to create synthetic data sets with varied autocovariance functions and biases. Across our data sets, the  window method favorably compromised bias sensitivity and discard time variability, and appeared reasonably robust. By increasing the variability of our data, we increased confidence that this applies to higher variance data (Section 4.3); by decreasing the autocorrelation (Section 4.4) we increased confidence that this applies to less correlated data. However, we acknowledge our data’s coverage of possible time series generated from molecular simulations remains limited.

The initial transients in our synthetic data decay quickly enough that most bias is eliminated by the end of a “standard” length binding free energy simulation. This is a sweet spot for testing truncation heuristics because the choice of truncation point is especially critical. We expect the data to be reasonably representative of absolute binding free energy calculations, but time series from other molecular simulations may not occupy this sweet spot. In the case where a rapidly decaying initial transient only biases the first few samples of a long time series, it is still important not to erroneously discard large fractions of the data (Section 4.1). Also, large but short-lived initial transients may have a fairly long-lived effect on cumulative averages if no truncation is performed. Therefore, the use of these heuristics is still justified. In the case where insufficient sampling has been performed and all data remain highly biased, these heuristics can still help to reduce bias. A key strength of these automated methods is that they can be applied without prior knowledge of the data. When few time series are generated or there is substantial prior knowledge of likely initial transient behavior, manual selection or fixed truncation points, respectively, may produce better results.

When applying generalized MSER methods to free energy calculations, a practical question is whether they should be applied to the overall time series (e.g., after combining all λ state gradients using thermodynamic integration), or applied individually to the gradients/perturbed energies from each window. As the optimal truncation points are expected to vary substantially between states, the former approach may reduce the quantity of data unnecessarily discarded, while the latter should reduce noise in the time series. We have not fully answered this question, but we have shown that the  window method appears to offer reasonable performance on synthetic data fit both to the overall stage free energy change (Table 2) and to a single noisy λ state (Section S6). These heuristics appear suitable for both approaches.

#### 4.6.2 Calculating Uncertainty

While inter-run uncertainty estimates are more robust,^52^ single-run uncertainty estimates are required in the absence of replicates. Flyvbjerg and Petersen argue for nonoverlapping block-averaging rather than directly using the autocovariance function to calculate uncertainty,^14^ while a current best practices guide for molecular simulation does not recommend either method over the other.^53^ We make a case for the use of the autocovariance function. Flyvbjerg and Petersen argue that the block-averaging method is cheaper than calculating the autocovariance function and avoids having to select the truncation point of the sum of autocovariance terms. However, using overlapping block averages provides a reduced variance variance estimate compared to nonoverlapping block averages, and is equivalent to applying an appropriately sized Bartlett window to the autocovariance function.^20,21^ This clarifies that the problem of truncation point selection is not avoided by using a block averaging method; it is closely related to the issue of block size selection. Furthermore, the additional computational cost (which is likely negligible with modern hardware) can bring a reduced variance variance estimate. As discussed by Gowers et al., use of the autocovariance function is also easily automatable.^54^ In addition, methods we are aware of for estimating the optimal block size require calculating the autocovariance function as an intermediate step,^23,55−57^ making more direct use of the autocovariance function simpler. Therefore, calculating variance of the mean estimates using autocovariance function-based methods may be preferable. Using uncorrelated or window estimators (without determining a suitable window size based on the data) may substantially underestimate the variance of the mean due to the incomplete treatment of correlation. Of the initial sequence methods, Geyer’s initial positive sequence method will often provide the smallest underestimate because it produces the latest truncation of the autocorrelation function. However, this will still systematically underestimate the variance of the mean, as noise in the autocorrelation function will result in early truncation (as negative Γ terms will appear earlier in the series with increasing noise). Therefore, approaches that fit the tail of the autocorrelation function to an exponential will likely provide superior uncertainty estimates.^27,48^

To illustrate the underestimation of uncertainty, we calculated the percentage of synthetic time series where the estimated 95% confidence intervals covered the true mean (the coverage) after truncation (Table S9). We did this for the “standard” free vanish stage synthetic ensembles, which did not contain initial transients. For the relatively uncorrelated T4L system, the coverage of the confidence intervals were very similar for all methods and close to the expected 95%. In contrast, for the more correlated PDE2a system, the coverages for all systems were well below 95%, even though there were still almost 1000 effectively uncorrelated samples. They were worst for the uncorrelated estimate (44%), and improved up to the  window method (71%) as autocorrelation was increasingly accounted for. Coverage then decreased slightly for the initial sequence methods (≈64%). We note that further downward bias can be introduced by truncating time series with MSER methods prior to calculating confidence intervals, as done here; by definition, MSER picks the truncation point which minimizes the estimated standard error. This bias will be greater for methods which are more prone to overdiscarding. As shown by eq 18, Chodera’s method of maximizing the effective sample size is closely related to MSER, so the issue of downward-biased uncertainty estimates also applies. When using generalized MSER or maximum effective sample size truncation metrics, users should be aware that they may be introducing additional downward bias to subsequent uncertainty estimates. This is without even considering that single runs often become stuck in local minima in configuration space, an issue which multiple runs may help to diagnose.

#### 4.6.3 Subsampling is Not Recommended

Producing better estimates with less information is impossible, so subsampling is not recommended when the cost of saving and using samples is negligible.^16^ Samples which are not fully correlated each contain new information (albeit less than uncorrelated samples), and discarding them adds noise to the variance and mean estimates. Subsampling may substantially increase the variance of the mean estimate and the variance estimate. Concretely, we show in Section S9 that when a sufficiently long stationary time series has a purely exponential correlation function with a half-life much greater than the sampling interval, subsampling at the interval of the statistical inefficiency increases the variance of the mean by 31%.

For this reason, we recommend against subsampling input data for the Bennett Acceptance Ratio (BAR) and the Multistate Bennett Acceptance Ratio (MBAR) free energy estimators.^58,59^ These are no longer the maximum likelihood free energy estimators when applied to correlated samples. Still, they remain robust estimators, and correcting for the correlation is unlikely to yield much improvement unless there are large differences in correlation between states.^60,61^ However, subsampling input for MBAR is often recommended because the asymptotic variance estimate given by Kong et al. and used in the original MBAR paper is derived for uncorrelated samples, meaning it is a large underestimate when there is substantial correlation.^59,62^ While subsampling decreases bias in this uncertainty estimate, it adds noise to the free energy and uncertainty estimates. A better approach may be to use an uncertainty estimator which accounts for correlation, for example, block bootstrapping,^23,57,63^ or a corrected asymptotic estimator.^61,64^

#### 4.6.4 Speed

The computational cost of the generalized MSER methods is likely negligible compared to the cost of generating the data. However, computational cost may become a consideration when the algorithm is run repeatedly, for example if applied to individual λ states in alchemical free energy calculations. In general, the computational cost increases with increasing treatment of correlation, as more terms in the autocovariance sum must be evaluated. Hence, the  window method also has the advantage of speed over the initial sequence methods. In our current implementation (RED 0.1.1),^65−67^ we generally found the ratio of speeds to be ≈1:2:8 for the Uncorrelated, Window Size , and all “Initial Sequence” methods, respectively on the “standard” time series of 10^4^ points. However, this is highly dependent on the implementation.

## 5 Conclusions

We have reformulated White’s MSER to provide a spectrum of truncation point selection heuristics which differ in their treatment of autocorrelation. These include a method effectively equivalent to Chodera’s maximum effective sample size heuristic.^2^ We tested these methods by generating ensembles of synthetic time series modeled on free energy change estimates from long absolute binding free energy calculations. Heuristics were assessed by their RMSE to the infinite-time ensemble average of the synthetic data.

We observed a general trade-off: methods which more thoroughly accounted for autocorrelation often showed late and variable truncation times; methods which less thoroughly accounted for autocorrelation often showed early truncation, relative to the optimal fixed-time truncation point. This increased variance and bias, respectively. We found that using a window estimator of the variance with size  provided a good compromise between these extremes, generally providing robust truncation point estimates. Rerunning our analyses on noisier and less correlated data produced similar conclusions.

All methods tested in this work are implemented in the open-source Python package RED (Robust Equilibration Detection, where equilibration is used in the sense of finding the optimal truncation point), available from the PyPI, conda-forge, and at github.com/fjclark/red. This provides alternatives for the PyMBAR timeseries “detect_equilibration” function. While these methods were useful for selecting truncation points given data, they were not generally useful for detecting insufficient sampling (lack of equilibration) in our data.

Future work may involve testing these heuristics on a broader range of synthetic data. In addition, further studies may focus on adapting these heuristics to multiple repeat runs (using globally centered autocovariances).^68,69^ This is likely a more robust approach and may reduce some of the issues we encountered with late truncation.

## Data Availability

All methods were implemented in the open-source Python package RED (robust equilibration detection, where equilibration is used in the sense of finding the optimal truncation point) available from the PyPI, conda-forge, and at github.com/fjclark/red. A complete workflow to reproduce the study beginning from the absolute binding free energy gradient data is provided at github.com/michellab/Robust-Equilibration-Detection-Paper and all data are provided on Zenodo.^51^

## Supporting Information Available

Detailed analysis of modeling the simulated data; detailed analysis and results for all generalized MSER heuristics on all synthetic ensembles; coverages of 95% confidence intervals for truncated data from the free vanish stage ensembles^51^ (PDF)
