# Supplementary material for: Robust Automated Truncation Point Selection for Molecular Simulations
Source: J Chem Theory Comput. 2024 Dec 23;21(1):88–101. doi: 10.1021/acs.jctc.4c01359 (PMC11736681; doi:10.1021/acs.jctc.4c01359)
Supplement: Supplementary file 1 — ct4c01359_si_001.pdf [file ct4c01359_si_001.pdf]

Supplementary Information for Robust Automated Truncation Point Selection for Molecular Simulations

Finlay Clark,*,† Daniel J. Cole,‡ and Julien Michel†

†*EaStCHEM School of Chemistry, University of Edinburgh, David Brewster Road, Edinburgh EH9 3FJ, United Kingdom*

‡*School of Natural and Environmental Sciences, Newcastle University, Newcastle upon Tyne NE1 7RU, United Kingdom*

E-mail: finlay.clark@ed.ac.uk

S1 Decomposition of RMSE into Bias and Variance Terms

It is well known that the overall RMSE can be decomposed into bias and variance terms.[1] For clarity, the full derivation is given here in our notation:

$$
\begin{aligned}
\mathrm{RMSE}_{\mathrm{Trajs}} &= \sqrt{\left\langle(\langle A\rangle_{[n_0,N]} - \langle A\rangle_\pi)^2\right\rangle_{\mathrm{Trajs}}} && \text{(S1)}\\
&= \sqrt{\left\langle \langle A\rangle^2_{[n_0,N]} - 2\langle A\rangle_{[n_0,N]}\langle A\rangle_\pi + \langle A\rangle^2_\pi \right\rangle_{\mathrm{Trajs}}} && \text{(S2)}\\
&= \sqrt{\left\langle \langle A\rangle^2_{[n_0,N]} \right\rangle_{\mathrm{Trajs}} - 2\left\langle\langle A\rangle_{[n_0,N]}\right\rangle_{\mathrm{Trajs}}\langle A\rangle_\pi + \langle A\rangle^2_\pi} && \text{(S3)}\\
&= \sqrt{\left\langle \langle A\rangle^2_{[n_0,N]} \right\rangle_{\mathrm{Trajs}} - \left\langle\langle A\rangle_{[n_0,N]}\right\rangle^2_{\mathrm{Trajs}} + \left\langle\langle A\rangle_{[n_0,N]}\right\rangle^2_{\mathrm{Trajs}} - 2\left\langle\langle A\rangle_{[n_0,N]}\right\rangle_{\mathrm{Trajs}}\langle A\rangle_\pi + \langle A\rangle^2_\pi} && \text{(S4)}\\
&= \sqrt{\left\langle \langle A\rangle^2_{[n_0,N]} - 2\langle\langle A\rangle_{[n_0,N]}\rangle_{\mathrm{Trajs}}\langle A\rangle_{[n_0,N]} + \langle\langle A\rangle_{[n_0,N]}\rangle^2_{\mathrm{Trajs.}} \right\rangle_{\mathrm{Trajs}} + (\langle\langle A\rangle_{[n_0,N]}\rangle_{\mathrm{Trajs}} - \langle A\rangle_\pi)^2} && \text{(S5)}\\
&= \sqrt{\left\langle(\langle A\rangle_{[n_0,N]} - \langle\langle A\rangle_{[n_0,N]}\rangle_{\mathrm{Trajs.}})^2\right\rangle_{\mathrm{Trajs}} + (\langle\langle A\rangle_{[n_0,N]}\rangle_{\mathrm{Trajs}} - \langle A\rangle_\pi)^2} && \text{(S6)}\\
&= \sqrt{\mathrm{Var}_{\mathrm{Trajs}} + \mathrm{Bias}^2_{\mathrm{Trajs}}}. && \text{(S7)}
\end{aligned}
$$

S2 Modelling the Bound Vanish Stages of the Absolute Binding Free Energy Data

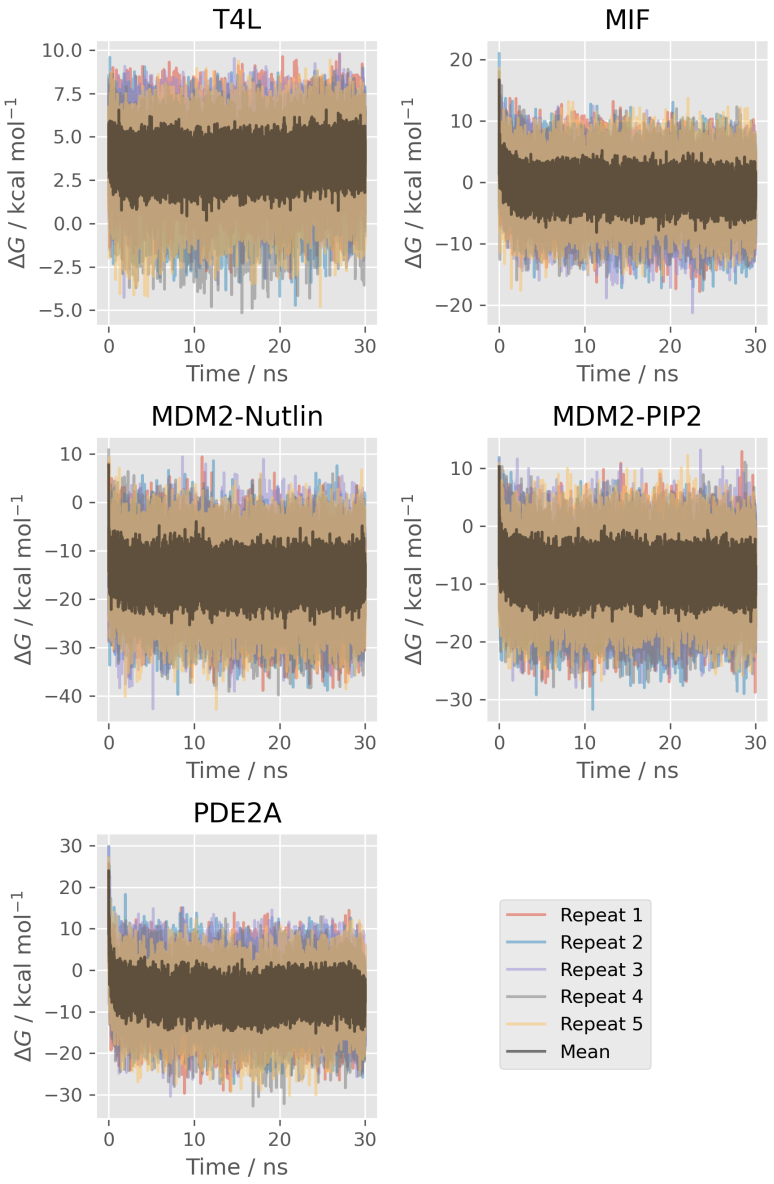

Figure S1: Time series of sampled free energy changes for all systems for bound vanish stages of the absolute binding free energy calculations. ΔG estimates obtained by integrating the gradients of the free energy over all windows using the trapezoidal rule.

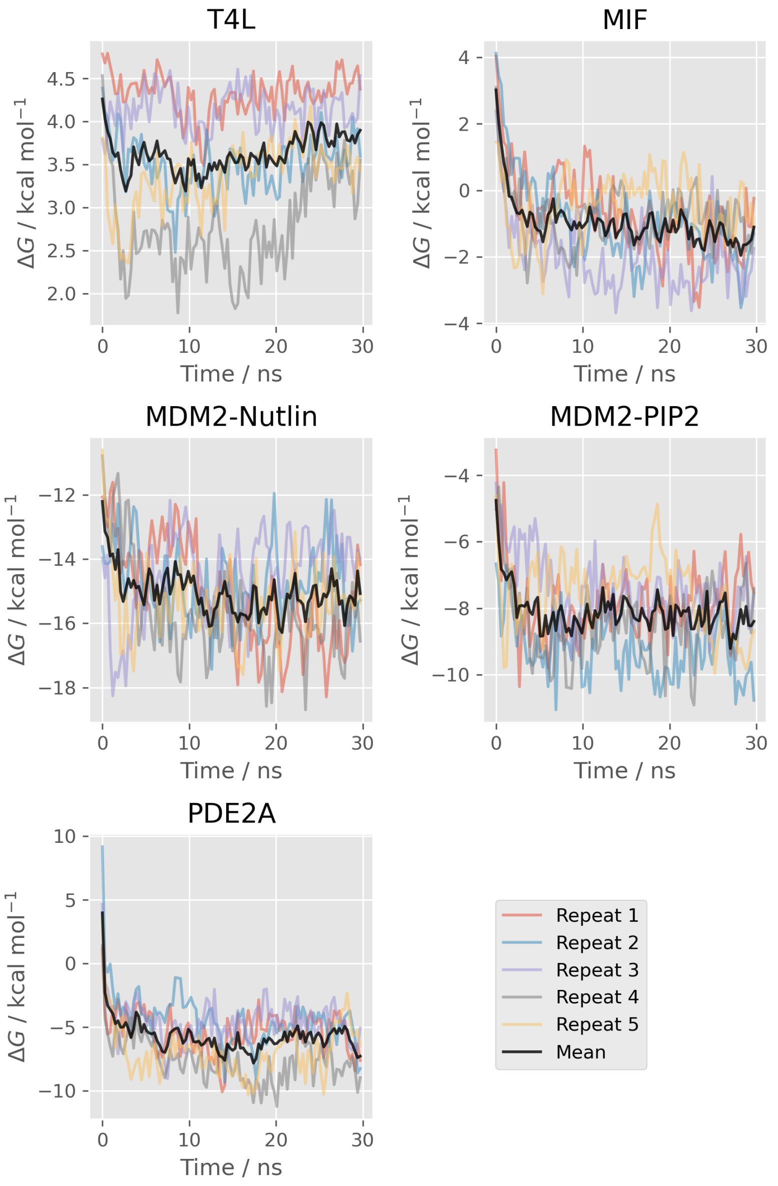

Figure S2: Block-averaged time series of sampled free energy changes for all systems for bound vanish stages of the absolute binding free energy calculations. Block averaging was performed using 100 blocks to more clearly show trends. ΔG estimates obtained by integrating the gradients of the free energy over all windows using the trapezoidal rule.

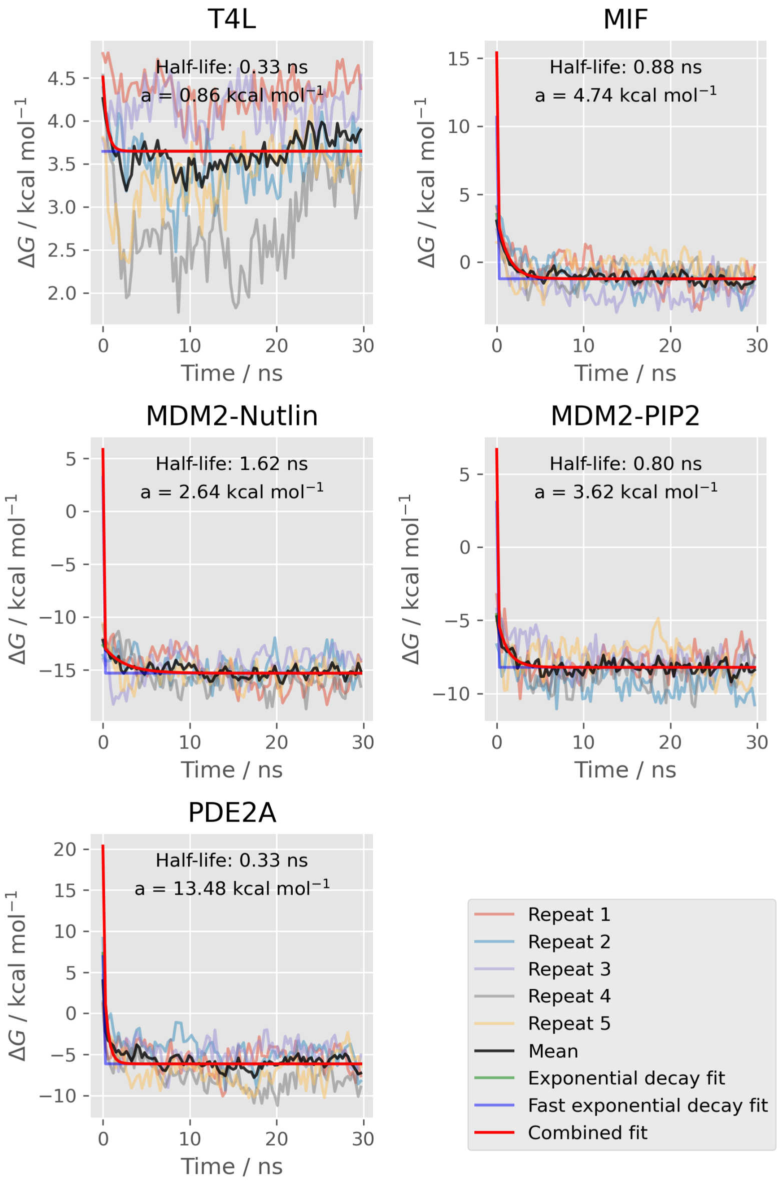

Figure S3: Exponential fits to the bound vanish leg time series of the absolute binding free energy calculations. The overall fit is composed of a “fast” and an initial exponential fit. Time series block averaged with 100 blocks to more clearly show trends.

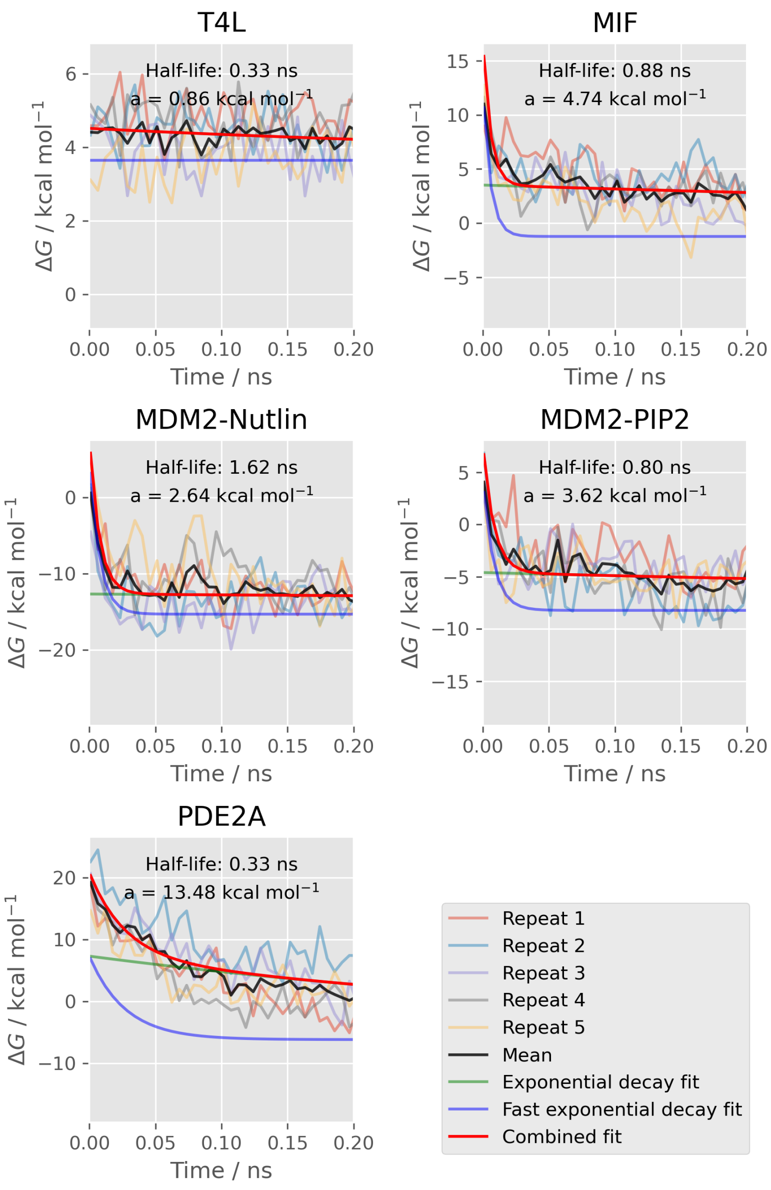

Figure S4: Exponential fits to the bound vanish leg time series of the absolute binding free energy calculations, zoomed in to show only the first 0.2 ns. In this region, the additional "fast" exponential fit is required to model the trend. Time series block averaged with 100 blocks to more clearly show trends.

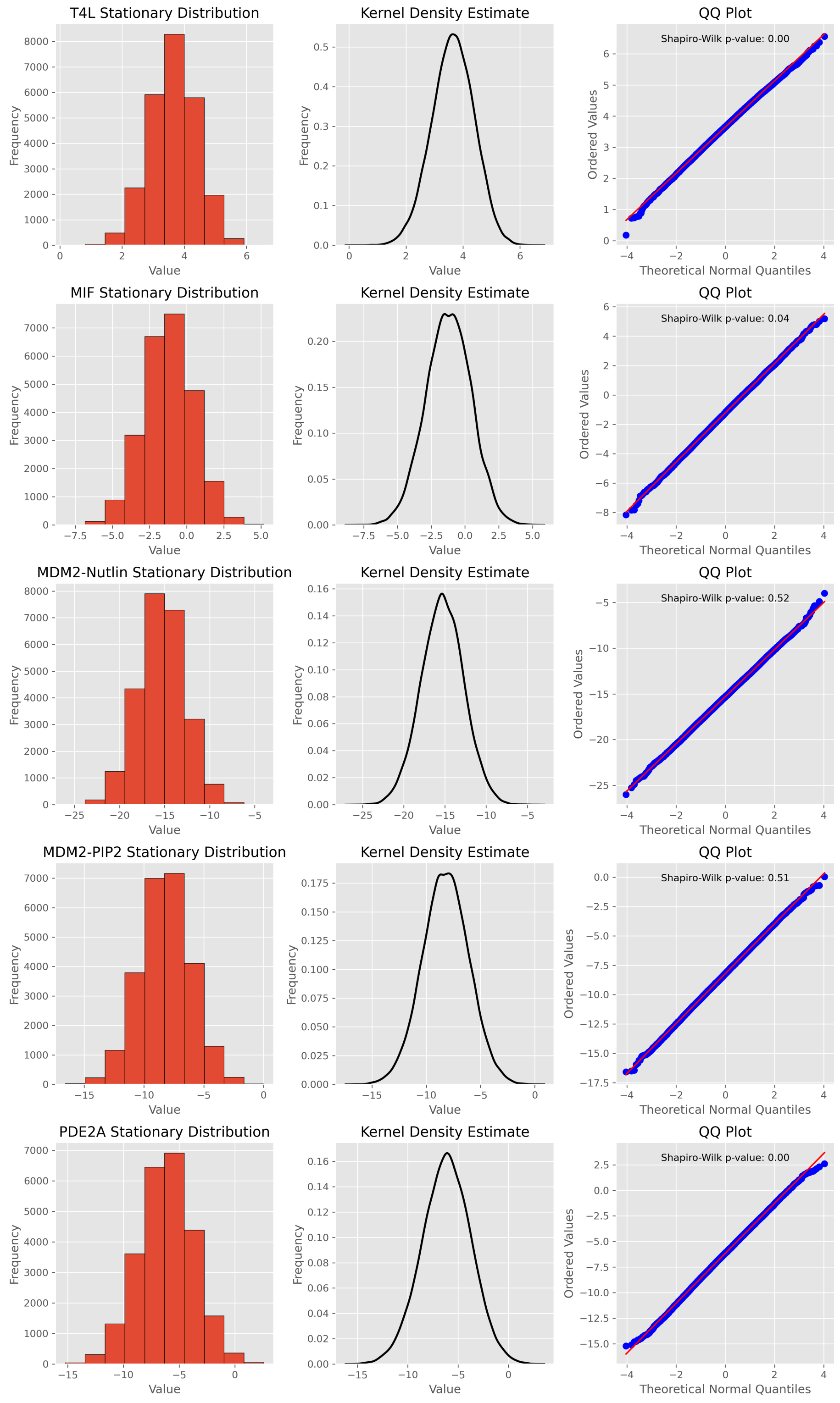

Figure S5: Histograms, kernel density estimates, and QQ plots of the distributions of ΔG estimates obtained over the last 20 ns of the bound vanish stages of the absolute binding free energy calculations. For some systems, the Shapiro-Wilk test showed significant evidence for non-normality of the distributions. However, the deviations were small, meaning that modelling the distributions as normal was reasonable.

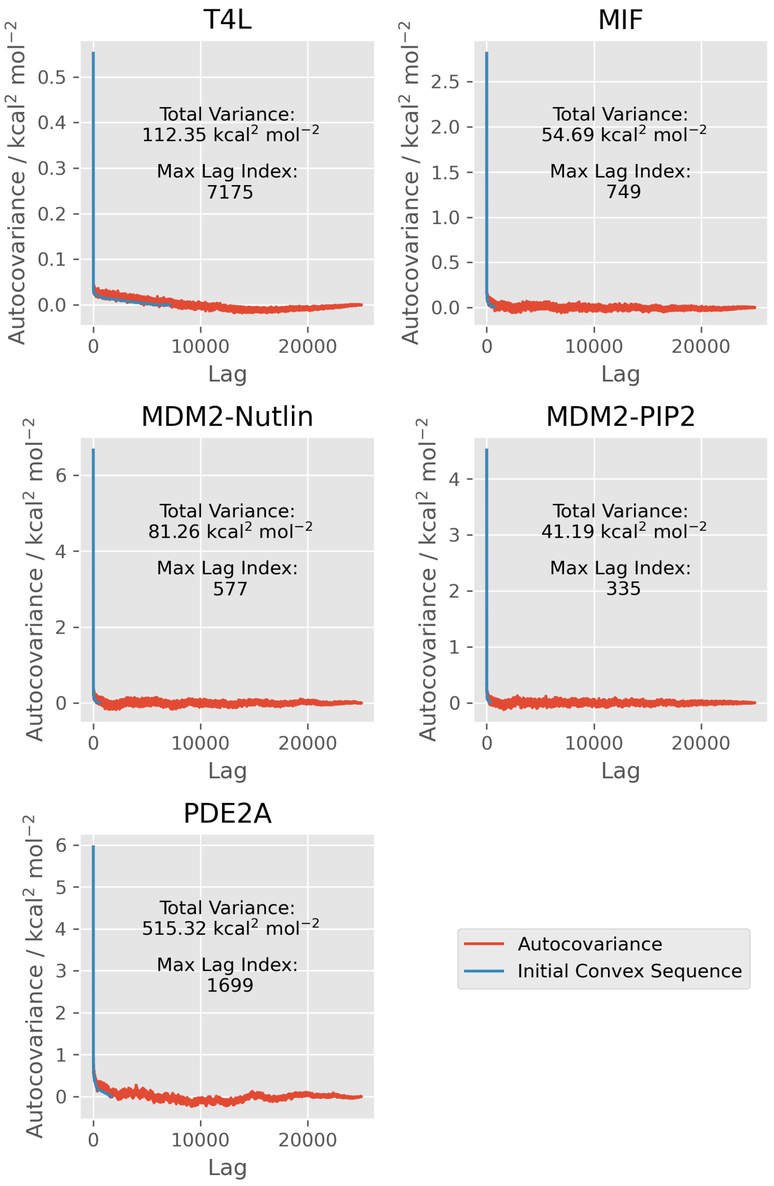

Figure S6: Estimated ("Autocovariance") and fitted autocovariance ("Initial Convex Sequence") functions obtained from the final 20 ns of the bound vanish stages of the absolute binding free energy calculations.

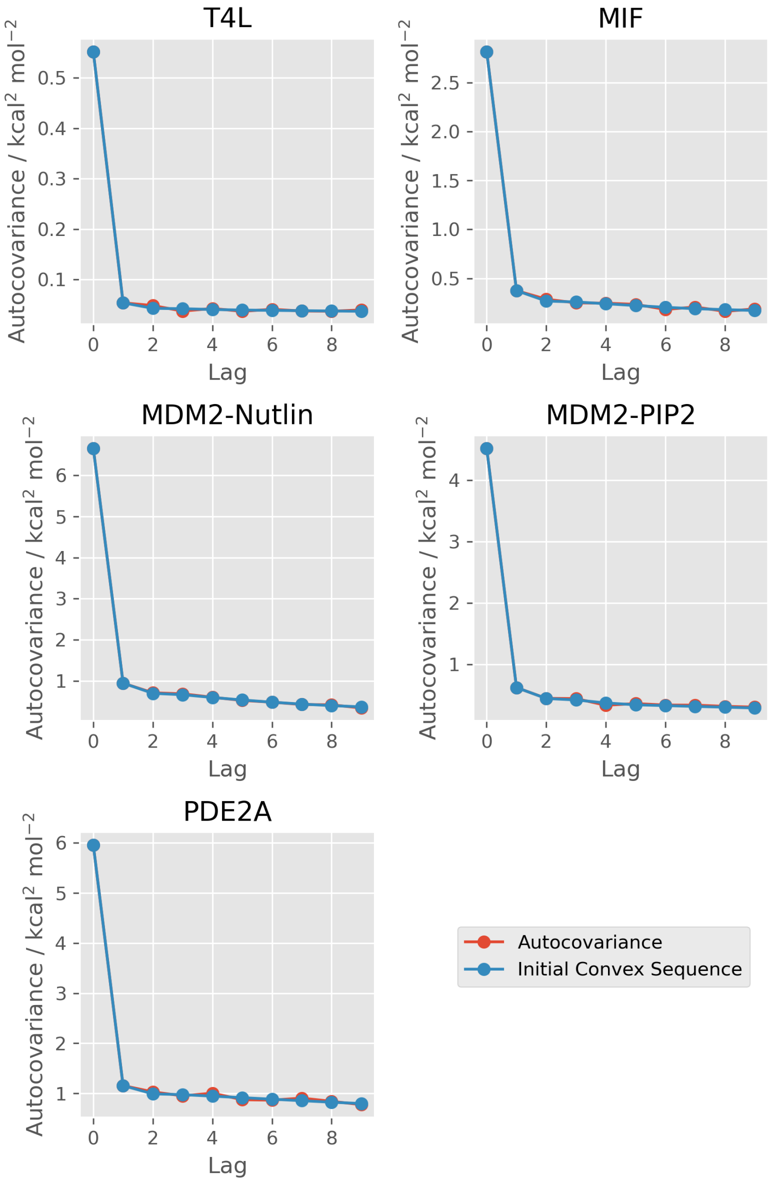

Figure S7: Early lag time estimated ("Autocovariance") and fitted autocovariance ("Initial Convex Sequence") functions obtained from the final 20 ns of the bound vanish stages of the absolute binding free energy calculations.

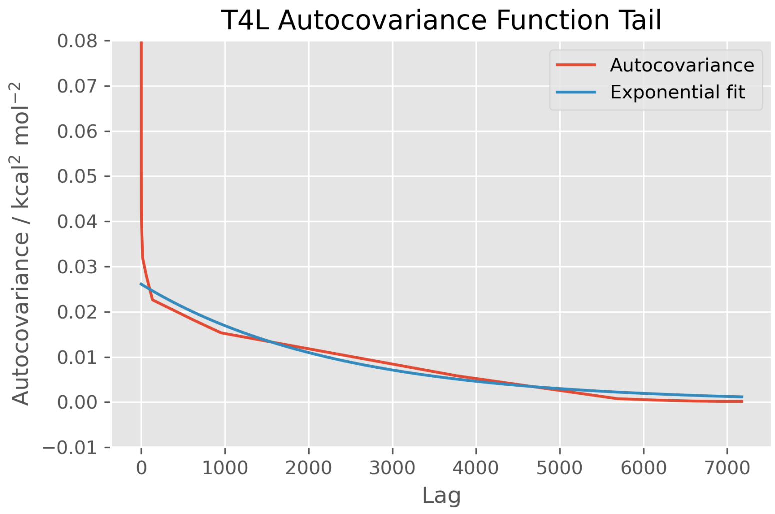

Figure S8: The long tail of the estimated T4L autocovariance function is reasonably well described by a relatively slow exponential decay. This is preceded by relatively fast decay at early lag times.

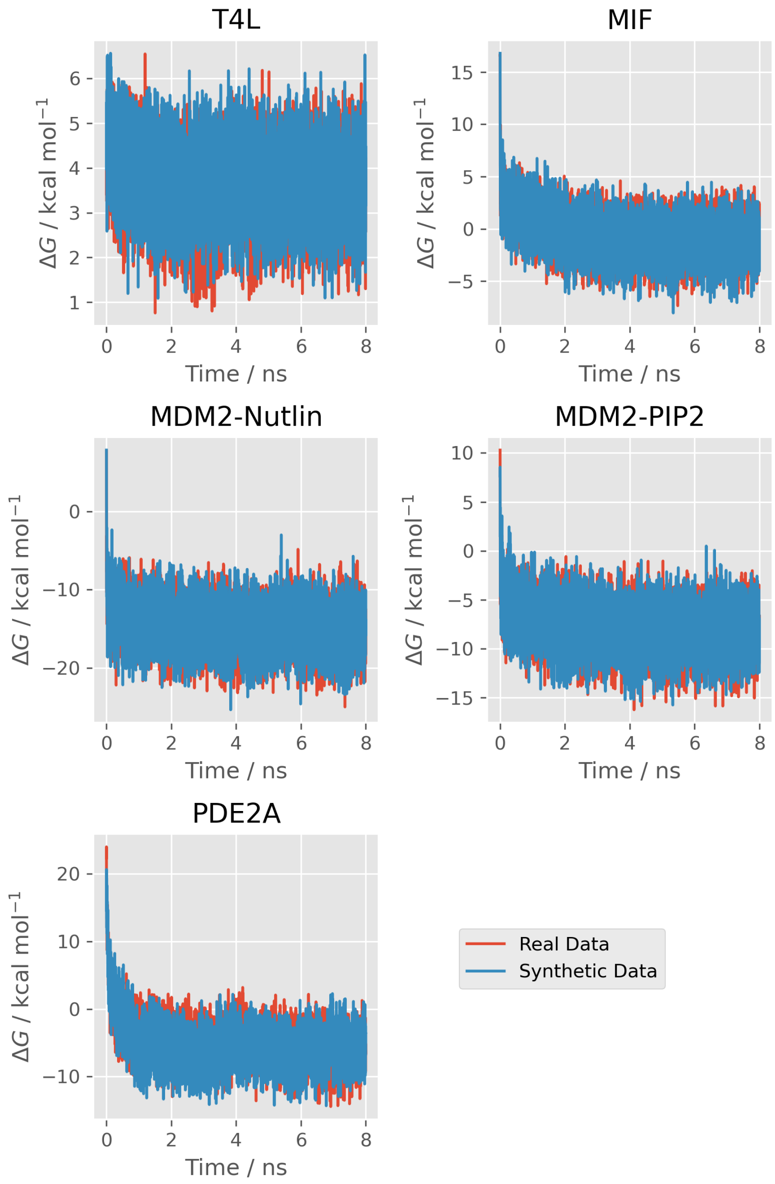

Figure S9: Random examples of synthetic ΔG time series against the time series from simulation they were fit to.

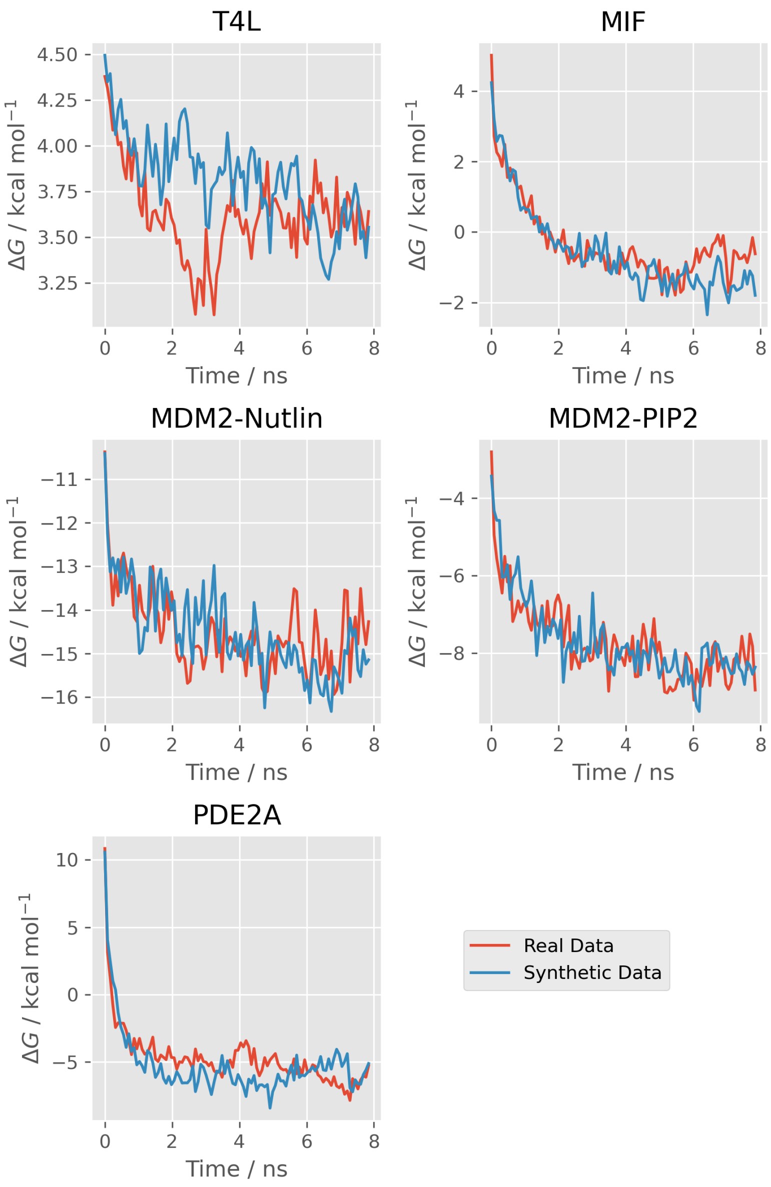

Figure S10: Random examples of synthetic ΔG time series against the time series from simulation they were fit to. Block averaged with 100 blocks to more clearly show trends and features.

S3 Modelling the Free Vanish Stages of the Absolute Binding Free Energy Data

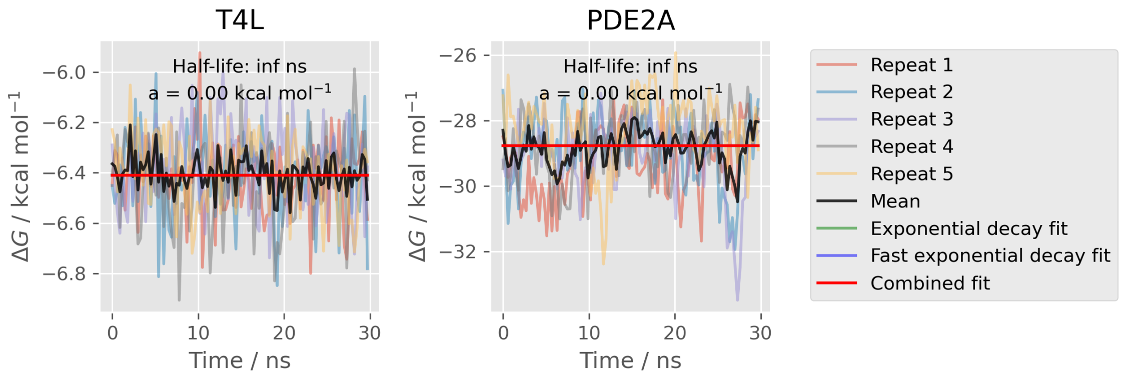

Figure S11: Exponential fits to the free vanish leg time series of the absolute binding free energy calculations. Time series block averaged with 100 blocks to more clearly show trends.

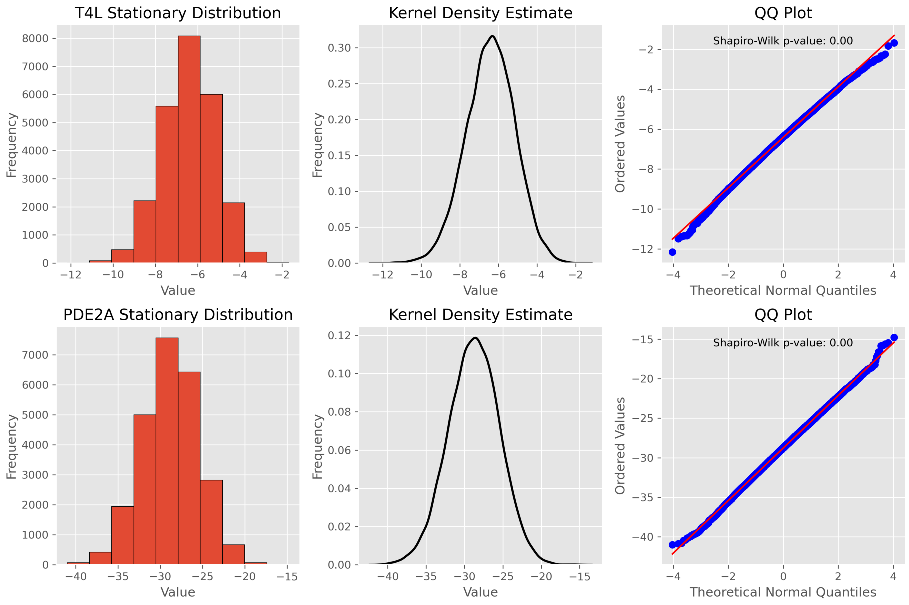

Figure S12: Histograms, kernel density estimates, and QQ plots of the distributions of ΔG estimates obtained over the last 20 ns of the free vanish stages of the absolute binding free energy calculations. For both systems, the Shapiro-Wilk test showed significant evidence for non-normality of the distributions. However, the deviations were small, meaning that modelling the distributions as normal was reasonable.

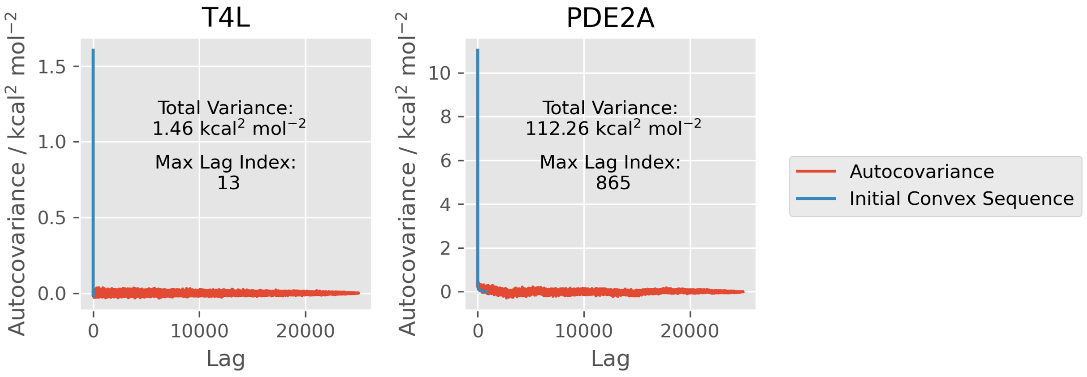

Figure S13: Estimated ("Autocovariance") and fitted autocovariance ("Initial Convex Sequence") functions obtained from the final 20 ns of the free vanish stages of the absolute binding free energy calculations.

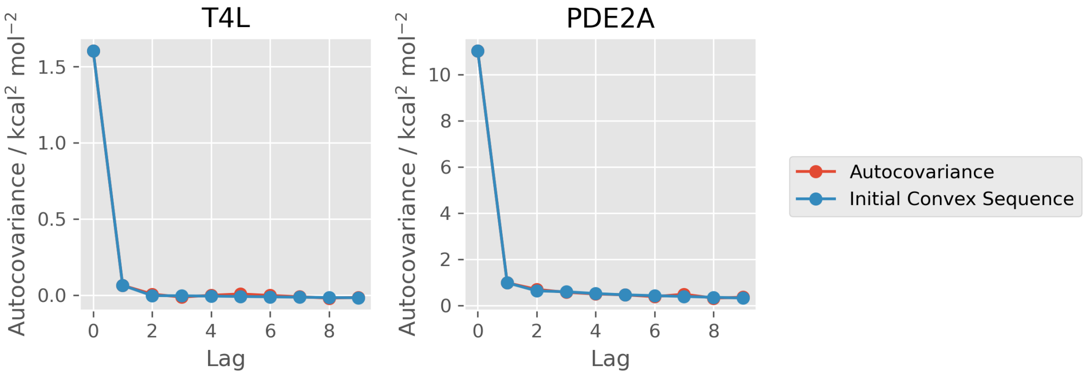

Figure S14: Early lag time estimated ("Autocovariance") and fitted autocovariance ("Initial Convex Sequence") functions obtained from the final 20 ns of the free vanish stages of the absolute binding free energy calculations.

Table S1: Model Parameters Fitted to Free Vanish Stages of Absolute Binding Free Energy Calculations[a]

	Half-life (ns)	a (kcal mol^{-1})	Fast Half-life (ns)	Fast a (kcal mol^{-1})	Total Variance (kcal^2 mol^{-2})	Max Lag Index
T4L	∞	0.00	∞	0.00	1.5	13
PDE2A	∞	0.00	∞	0.00	110	865

[a] Total variance refers to the total variance of the mean, obtained by summing the autocovariance series from - max lag index to + max lag index, where the series and maximum lag indices were estimated according to Geyer's initial convex sequence rules. "a" refers to the pre-exponential factors.

S4 Performance of Truncation Heuristics on Free Vanish Leg Time Series

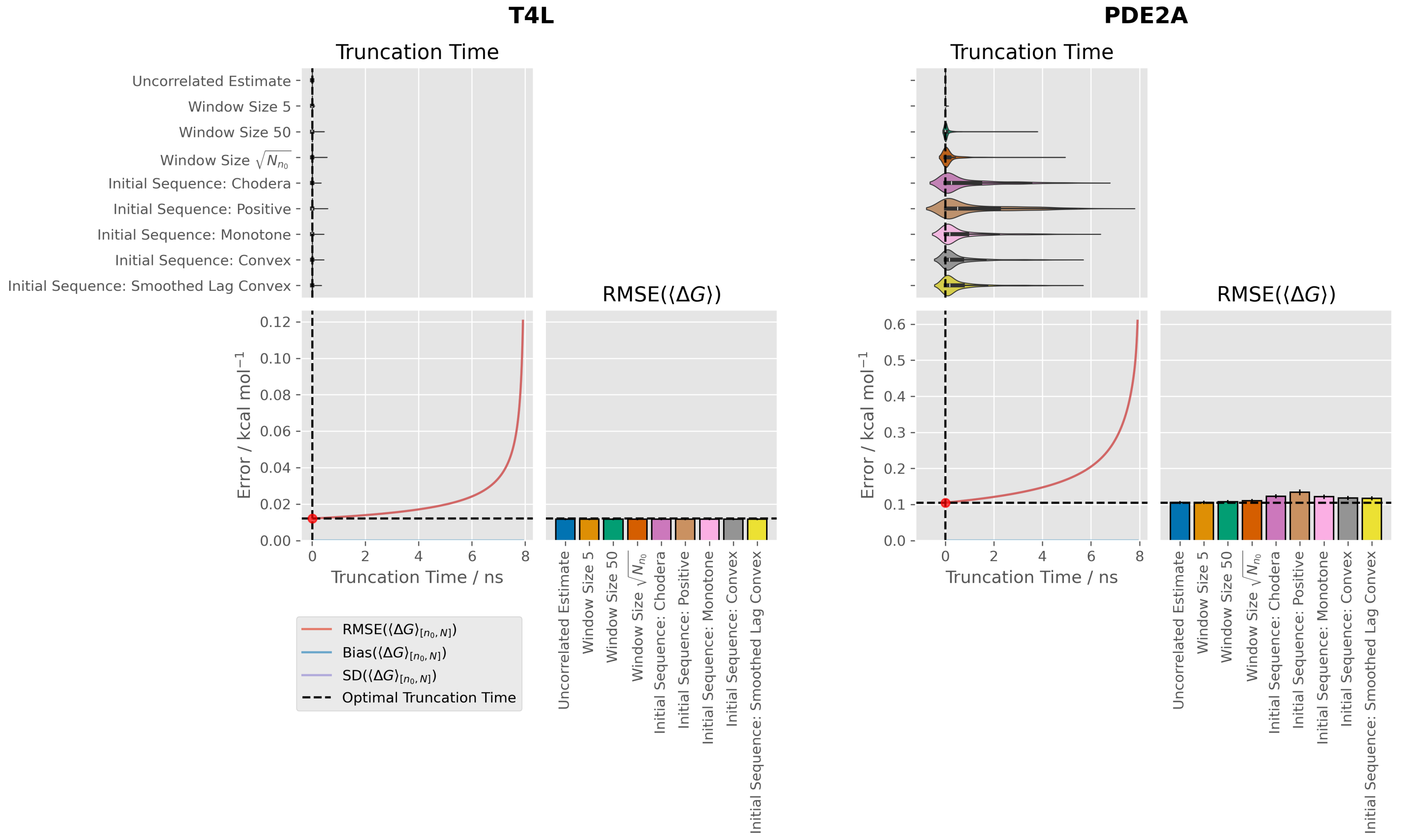

Figure S15: Discard times, RMSEs, and underlying time series properties for the free vanish stage time series for T4L and PDE2A. The top panels show kernel density estimates of the distributions of times discarded with each method. The bottom left panels show the RMSEs which would be obtained with an infinitely large ensemble of synthetic time series with fixed truncation points. The red dot indicates the optimum fixed-time truncation point. This is at 0 as there are no biases. The right panels show the RMSEs obtained with each generalised MSER method.

Table S2: Ensemble RMSEs for all Generalised MSER Heuristics for the Free Vanish Data[a]

Method	T4L	PDE2A
Uncorrelated Estimate	$0.0117^{0.0123}_{0.0112}$	$0.105^{0.110}_{0.101}$
Window Size 5	$0.0117^{0.0123}_{0.0112}$	$0.105^{0.110}_{0.101}$
Window Size 50	$0.0118^{0.0123}_{0.0112}$	$0.107^{0.112}_{0.103}$
Window Size $\sqrt{N_{n_0}}$	$0.0118^{0.0123}_{0.0112}$	$0.110^{0.115}_{0.105}$
Initial Sequence: Chodera	$0.0118^{0.0123}_{0.0112}$	$0.122^{0.128}_{0.117}$
Initial Sequence: Positive	$0.0118^{0.0123}_{0.0112}$	$0.134^{0.142}_{0.126}$
Initial Sequence: Monotone	$0.0118^{0.0123}_{0.0112}$	$0.121^{0.128}_{0.116}$
Initial Sequence: Convex	$0.0118^{0.0123}_{0.0112}$	$0.118^{0.123}_{0.112}$
Initial Sequence: Smoothed Lag Convex	$0.0118^{0.0123}_{0.0112}$	$0.117^{0.122}_{0.111}$

[a] All values in kcal mol^{-1}. Uncertainties are 95 % confidence intervals obtained by bootstrapping over 10000 iterations with replacement.

S5 Investigation of Adaptive Integration Scheme with the Max ESS Heuristic on PDE2a Free Vanish Leg Time Series

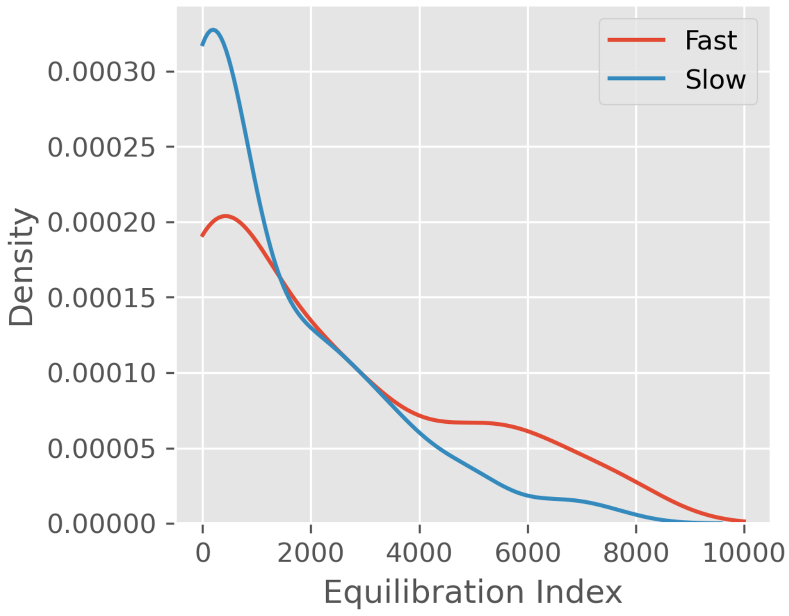

Figure S16: Kernel density estimate plot of truncation times selected with Chodera's maximum effective sample size heuristic (as implemented in PyMBAR's timeseries module) with and without the adaptive integration scheme described by Chodera et al. .[1–3] These synthetic trajectories have no bias and hence the optimum truncation time is at index 0. The heuristics were tested on the first 100 synthetic trajectories. The use of the adaptive integration scheme produced more erroneously late truncation times. The final index shown corresponds to 8 ns of simulation data.

S6 Performance of Truncation Heuristics on All Bound Vanish Leg Time Series Ensembles

Note that in some cases, the relatively low RMSE of the "Window Size 50" method on the block averaged data results from the large block size preventing late truncation when there are few data points (e.g. a window size of 50 requires more than 50 points, which is half of the time series length).

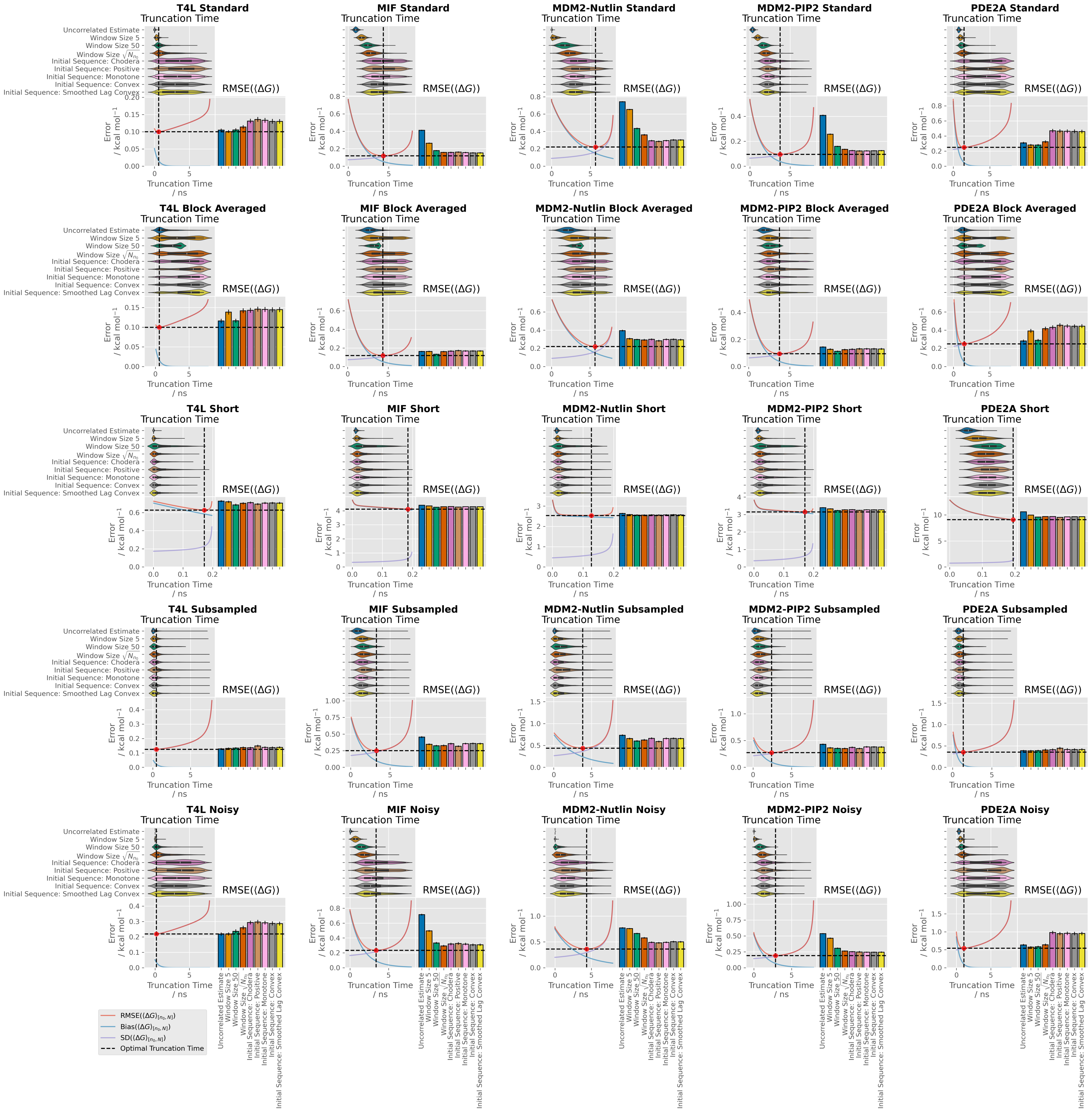

Figure S17: Discard times, RMSEs, and underlying time series properties for the bound vanish stage time series for all data sets. The top panels show kernel density estimates of the distributions of times discarded with each method. The bottom left panels show the RMSEs which would be obtained with an infinitely large ensemble of synthetic time series with fixed truncation points. The red dot indicates the optimum fixed-time truncation point.The right panels show the ensemble average RMSEs obtained with each of the truncation heuristics. Error bars are 95 % confidence intervals obtained by bootstrapping over 1000 iterations with replacement.

Table S3: Ensemble RMSEs for all Generalised MSER Heuristics for the “Noisy” Bound Vanish Data[a]

Method	T4L	MIF	MDM2-Nutlin	MDM2-PIP2	PDE2A
Uncorrelated Estimate	$0.218^{0.228}_{0.207}$	$0.715^{0.726}_{0.703}$	$0.771^{0.783}_{0.759}$	$0.536^{0.544}_{0.527}$	$0.634^{0.659}_{0.609}$
Window Size 5	$0.219^{0.229}_{0.209}$	$0.495^{0.508}_{0.483}$	$0.757^{0.769}_{0.745}$	$0.464^{0.473}_{0.454}$	$0.573^{0.597}_{0.550}$
Window Size 50	$0.236^{0.249}_{0.224}$	$0.333^{0.345}_{0.321}$	$0.663^{0.677}_{0.649}$	$0.307^{0.317}_{0.297}$	$0.584^{0.608}_{0.559}$
Window Size $\sqrt{N_{n_0}}$	$0.260^{0.273}_{0.248}$	$0.293^{0.305}_{0.281}$	$0.578^{0.593}_{0.564}$	$0.262^{0.271}_{0.252}$	$0.639^{0.671}_{0.608}$
Initial Sequence: Chodera	$0.293^{0.306}_{0.281}$	$0.318^{0.335}_{0.302}$	$0.491^{0.507}_{0.475}$	$0.251^{0.261}_{0.242}$	$0.980^{1.028}_{0.932}$
Initial Sequence: Positive	$0.297^{0.310}_{0.284}$	$0.326^{0.343}_{0.309}$	$0.481^{0.497}_{0.465}$	$0.248^{0.262}_{0.235}$	$0.948^{0.995}_{0.902}$
Initial Sequence: Monotone	$0.292^{0.305}_{0.279}$	$0.317^{0.334}_{0.300}$	$0.492^{0.508}_{0.477}$	$0.243^{0.253}_{0.234}$	$0.956^{1.004}_{0.908}$
Initial Sequence: Convex	$0.287^{0.300}_{0.275}$	$0.308^{0.324}_{0.292}$	$0.504^{0.519}_{0.489}$	$0.244^{0.253}_{0.234}$	$0.949^{0.998}_{0.902}$
Initial Sequence: Smoothed Lag Convex	$0.286^{0.299}_{0.273}$	$0.308^{0.324}_{0.293}$	$0.502^{0.517}_{0.487}$	$0.244^{0.254}_{0.235}$	$0.951^{1.000}_{0.904}$

[a] All values in kcal mol^{-1}. Uncertainties are 95 % confidence intervals obtained by bootstrapping over 10000 iterations with replacement.

Table S4: Ensemble RMSEs for all Generalised MSER Heuristics for the “Subsampled” Bound Vanish Data[a]

Method	T4L	MIF	MDM2-Nutlin	MDM2-PIP2	PDE2A
Uncorrelated Estimate	$0.128^{0.134}_{0.123}$	$0.454^{0.468}_{0.439}$	$0.732^{0.750}_{0.714}$	$0.428^{0.442}_{0.415}$	$0.388^{0.405}_{0.371}$
Window Size 5	$0.132^{0.137}_{0.126}$	$0.346^{0.359}_{0.333}$	$0.657^{0.677}_{0.637}$	$0.363^{0.377}_{0.348}$	$0.386^{0.403}_{0.370}$
Window Size 50	$0.133^{0.139}_{0.128}$	$0.325^{0.337}_{0.313}$	$0.600^{0.619}_{0.581}$	$0.353^{0.367}_{0.339}$	$0.388^{0.406}_{0.370}$
Window Size $\sqrt{N_{n_0}}$	$0.137^{0.144}_{0.131}$	$0.328^{0.340}_{0.315}$	$0.626^{0.646}_{0.605}$	$0.351^{0.366}_{0.337}$	$0.405^{0.434}_{0.380}$
Initial Sequence: Chodera	$0.136^{0.142}_{0.129}$	$0.356^{0.369}_{0.342}$	$0.662^{0.683}_{0.642}$	$0.371^{0.386}_{0.357}$	$0.411^{0.440}_{0.387}$
Initial Sequence: Positive	$0.149^{0.157}_{0.142}$	$0.317^{0.329}_{0.304}$	$0.589^{0.610}_{0.568}$	$0.349^{0.365}_{0.334}$	$0.445^{0.476}_{0.416}$
Initial Sequence: Monotone	$0.138^{0.145}_{0.132}$	$0.357^{0.370}_{0.343}$	$0.659^{0.679}_{0.639}$	$0.381^{0.395}_{0.367}$	$0.417^{0.446}_{0.391}$
Initial Sequence: Convex	$0.138^{0.144}_{0.131}$	$0.358^{0.371}_{0.345}$	$0.662^{0.682}_{0.642}$	$0.379^{0.393}_{0.365}$	$0.414^{0.444}_{0.388}$
Initial Sequence: Smoothed Lag Convex	$0.139^{0.145}_{0.132}$	$0.355^{0.368}_{0.342}$	$0.658^{0.678}_{0.638}$	$0.378^{0.392}_{0.364}$	$0.416^{0.445}_{0.390}$

[a] All values in kcal mol^{-1}. Uncertainties are 95 % confidence intervals obtained by bootstrapping over 10000 iterations with replacement.

Table S5: Ensemble RMSEs for all Generalised MSER Heuristics for the “Block Averaged” Bound Vanish Data[a]

Method	T4L	MIF	MDM2-Nutlin	MDM2-PIP2	PDE2A
Uncorrelated Estimate	$0.116^{0.121}_{0.110}$	$0.162^{0.168}_{0.156}$	$0.395^{0.403}_{0.386}$	$0.144^{0.149}_{0.140}$	$0.280^{0.295}_{0.266}$
Window Size 5	$0.138^{0.145}_{0.132}$	$0.162^{0.169}_{0.155}$	$0.306^{0.316}_{0.296}$	$0.128^{0.134}_{0.122}$	$0.391^{0.412}_{0.370}$
Window Size 50	$0.116^{0.121}_{0.111}$	$0.133^{0.138}_{0.128}$	$0.297^{0.304}_{0.289}$	$0.113^{0.118}_{0.108}$	$0.289^{0.301}_{0.276}$
Window Size $\sqrt{N_{n_0}}$	$0.142^{0.148}_{0.136}$	$0.161^{0.168}_{0.154}$	$0.292^{0.302}_{0.283}$	$0.125^{0.132}_{0.118}$	$0.417^{0.438}_{0.395}$
Initial Sequence: Chodera	$0.143^{0.149}_{0.137}$	$0.167^{0.174}_{0.159}$	$0.298^{0.308}_{0.288}$	$0.128^{0.135}_{0.122}$	$0.430^{0.451}_{0.409}$
Initial Sequence: Positive	$0.146^{0.152}_{0.140}$	$0.172^{0.180}_{0.164}$	$0.282^{0.293}_{0.272}$	$0.131^{0.139}_{0.124}$	$0.454^{0.475}_{0.433}$
Initial Sequence: Monotone	$0.145^{0.151}_{0.139}$	$0.167^{0.175}_{0.160}$	$0.298^{0.308}_{0.288}$	$0.131^{0.138}_{0.125}$	$0.444^{0.465}_{0.423}$
Initial Sequence: Convex	$0.144^{0.151}_{0.138}$	$0.167^{0.175}_{0.159}$	$0.299^{0.309}_{0.289}$	$0.131^{0.137}_{0.124}$	$0.443^{0.464}_{0.422}$
Initial Sequence: Smoothed Lag Convex	$0.145^{0.151}_{0.138}$	$0.167^{0.175}_{0.159}$	$0.294^{0.304}_{0.284}$	$0.130^{0.137}_{0.123}$	$0.443^{0.464}_{0.422}$

[a] All values in kcal mol^{-1}. Uncertainties are 95 % confidence intervals obtained by bootstrapping over 10000 iterations with replacement.

Table S6: Ensemble RMSEs for all Generalised MSER Heuristics for the “Short” Bound Vanish Data[a]

Method	T4L	MIF	MDM2-Nutlin	MDM2-PIP2	PDE2A
Uncorrelated Estimate	$0.727^{0.738}_{0.716}$	$4.397^{4.418}_{4.377}$	$2.637^{2.664}_{2.609}$	$3.393^{3.416}_{3.370}$	$10.594^{10.650}_{10.538}$
Window Size 5	$0.719^{0.730}_{0.708}$	$4.347^{4.368}_{4.326}$	$2.578^{2.606}_{2.549}$	$3.328^{3.352}_{3.304}$	$9.970^{10.033}_{9.910}$
Window Size 50	$0.686^{0.698}_{0.674}$	$4.251^{4.276}_{4.225}$	$2.554^{2.589}_{2.521}$	$3.233^{3.262}_{3.205}$	$9.586^{9.645}_{9.526}$
Window Size $\sqrt{N_{n_0}}$	$0.703^{0.714}_{0.691}$	$4.290^{4.312}_{4.267}$	$2.557^{2.588}_{2.527}$	$3.269^{3.295}_{3.243}$	$9.669^{9.731}_{9.606}$
Initial Sequence: Chodera	$0.710^{0.722}_{0.699}$	$4.301^{4.324}_{4.278}$	$2.566^{2.598}_{2.534}$	$3.283^{3.310}_{3.257}$	$9.671^{9.734}_{9.609}$
Initial Sequence: Positive	$0.694^{0.706}_{0.683}$	$4.267^{4.292}_{4.242}$	$2.559^{2.592}_{2.525}$	$3.245^{3.274}_{3.216}$	$9.534^{9.597}_{9.472}$
Initial Sequence: Monotone	$0.705^{0.717}_{0.694}$	$4.286^{4.310}_{4.262}$	$2.567^{2.599}_{2.535}$	$3.271^{3.299}_{3.244}$	$9.622^{9.683}_{9.560}$
Initial Sequence: Convex	$0.707^{0.718}_{0.695}$	$4.293^{4.317}_{4.269}$	$2.572^{2.604}_{2.541}$	$3.274^{3.302}_{3.248}$	$9.645^{9.708}_{9.582}$
Initial Sequence: Smoothed Lag Convex	$0.706^{0.717}_{0.694}$	$4.292^{4.315}_{4.269}$	$2.568^{2.600}_{2.536}$	$3.273^{3.300}_{3.246}$	$9.645^{9.708}_{9.584}$

[a] All values in kcal mol^{-1}. Uncertainties are 95 % confidence intervals obtained by bootstrapping over 10000 iterations with replacement.

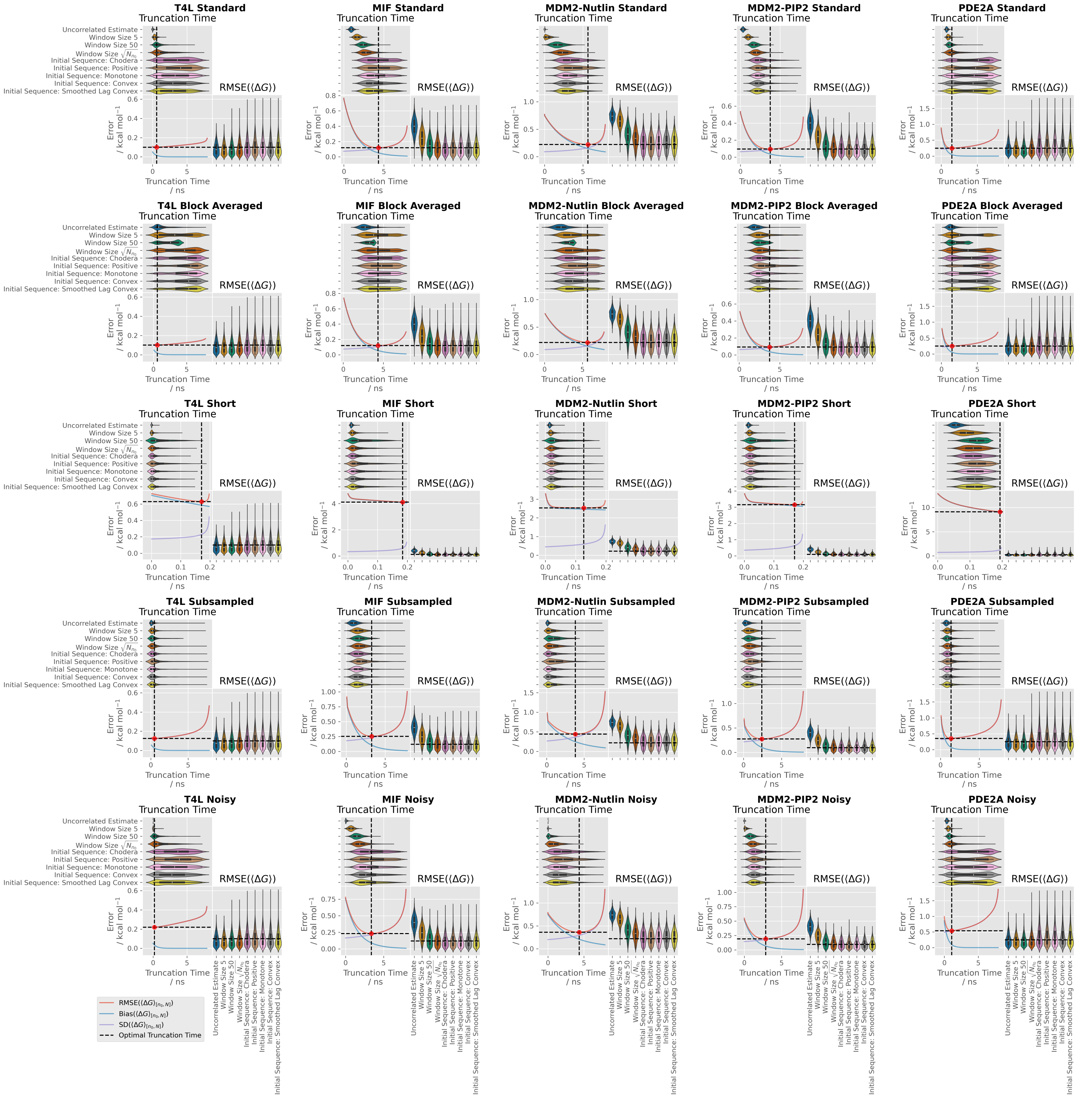

Figure S18: Discard times, unsigned errors, and underlying time series properties for the bound vanish stage time series for all data sets. The top panels show kernel density estimates of the distributions of times discarded with each method. The bottom left panels show the RMSEs which would be obtained with an infinitely large ensemble of synthetic time series with fixed truncation points. The red dot indicates the optimum fixed-time truncation point. The right panels show the distributions of unsigned errors obtained over the synthetic ensembles.

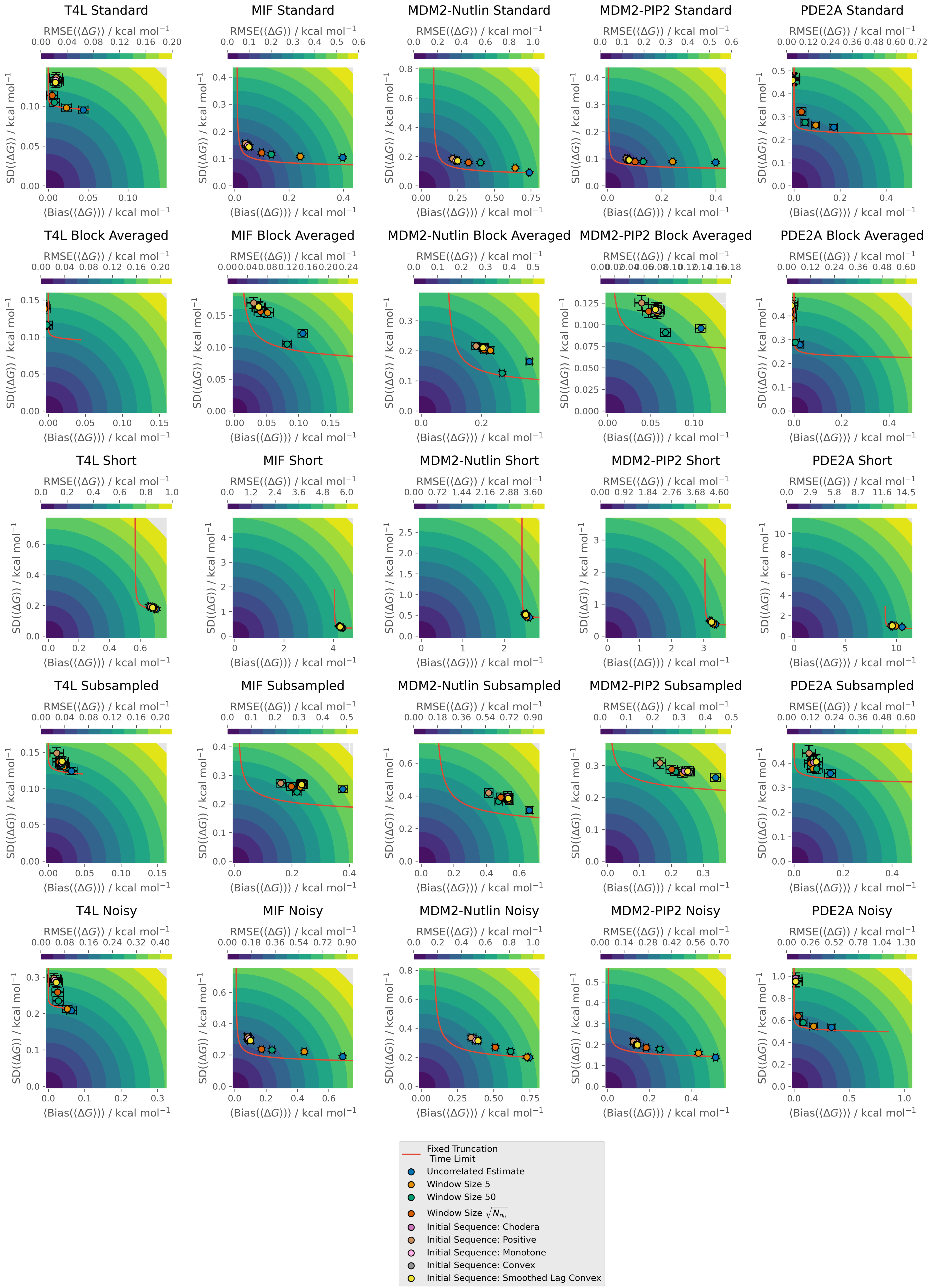

Figure S19: Decomposition of errors made by generalised MSER methods over ensembles of synthetic trajectories. Red lines show the fixed truncation point limits. Error bars are 95 % confidence intervals obtained by bootstrapping over 1000 iterations with replacement.

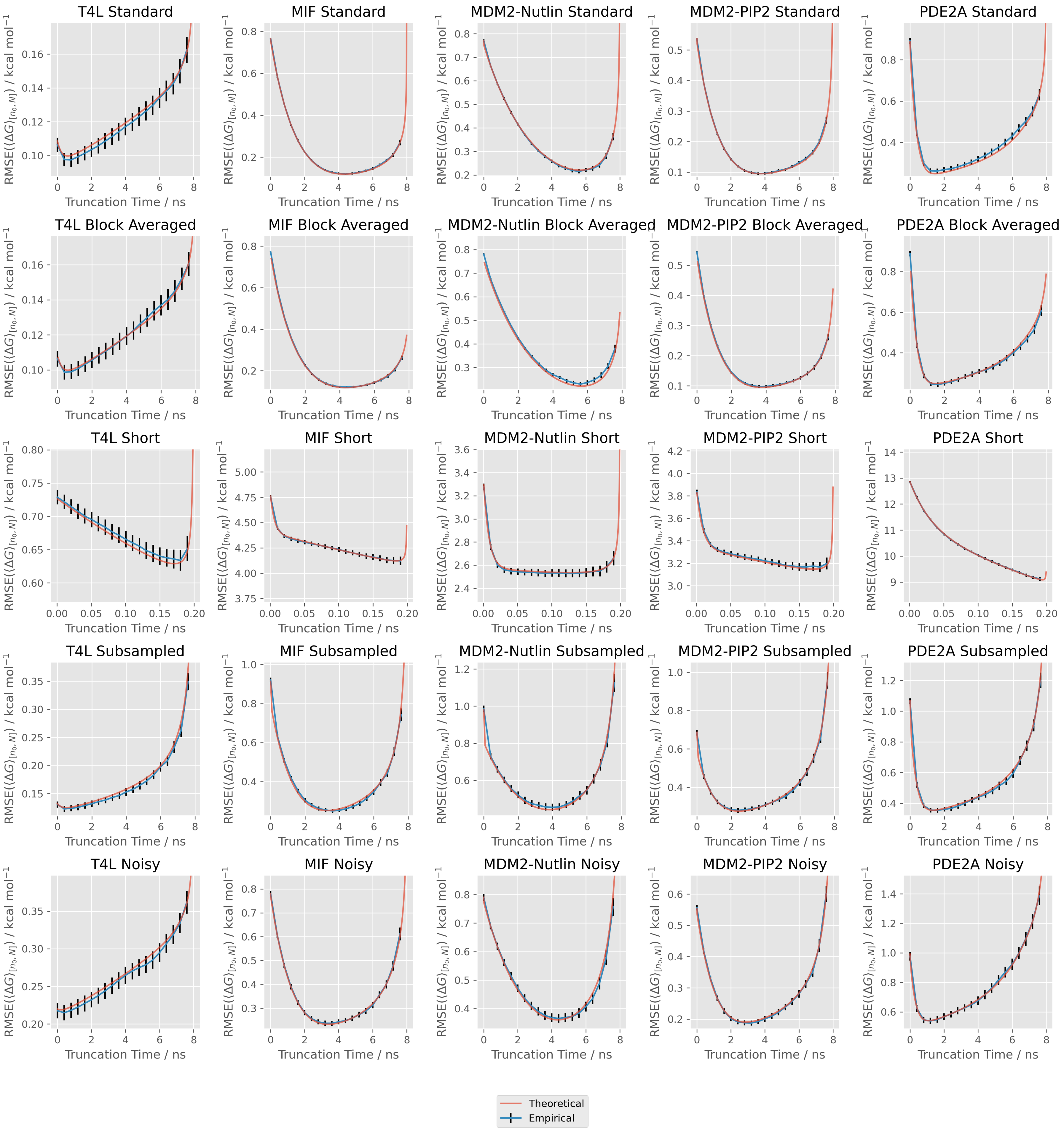

Figure S20: Theoretical versus empirical RMSEs obtained for all synthetic time series ensembles for all systems. Error bars are 95 % confidence intervals obtained by bootstrapping over 1000 iterations with replacement.

S7 Performance of Heuristics on Synthetic Data Modelled on Single λ State

To test the applicability of the methods to single λ states, rather than a free energy change integrated over all states, we modelled synthetic data on a single λ state. We chose the bound vanish $\lambda = 0.45$ state, which tends to show high noise and a substantial initial transient. Rather than integrating over all states using the trapezoidal rule, this window was given a weight of 1. Otherwise, the synthetic data were fit using the same procedure as for the "standard" synthetic ensembles. This generally produced greater ratios of total variance to the slow preexponential factor (Table S7) compared to the "standard" synthetic data (Table 1).

Table S7: Model Parameters Fitted to $\lambda = 0.45$ Bound Vanish Window of Absolute Binding Free Energy Calculations[a]

	Half-life (ns)	a (kcal mol^{-1})	Fast Half-life (ns)	Fast a (kcal mol^{-1})	Total Variance (kcal^2 mol^{-2})	Max Lag Index
T4L	0.16	2.5	∞	0	5000	4995
MIF	3.2	19	0.70	0.45	7100	859
MDM2-Nutlin	3.1	12	∞	0	29000	1475
MDM2-PIP2	3.0	11	∞	0	6500	1023
PDE2A	0.27	43	∞	0	49000	2231

[a] Total variance refers to the total variance of the mean, obtained by summing the autocovariance series from - max lag index to + max lag index, where the series and maximum lag indices were estimated according to Geyer's initial convex sequence rules. "a" refers to the pre-exponential factors.

Similarly to the "standard" synthetic ensembles, the methods which less thoroughly account for autocorrelation select earlier truncation times and perform better on systems with earlier optimal truncation times, and vice versa (Table S8 and Figures S21 and S22). As with the "standard" ensembles, the $\sqrt{N_{n_0}}$ window method appeared to strike a reasonable compromise between bias sensitivity and truncation time variability.

Table S8: Ensemble RMSEs for all Generalised MSER Heuristics for the Single Window Bound Vanish Data[a]

Method	T4L	MIF	MDM2-Nutlin	MDM2-PIP2	PDE2A
Uncorrelated Estimate	$0.68^{0.71}_{0.65}$	$9.00^{9.06}_{8.95}$	$5.79^{5.90}_{5.69}$	$5.21^{5.26}_{5.16}$	$2.41^{2.50}_{2.31}$
Window Size 5	$0.69^{0.72}_{0.66}$	$8.15^{8.22}_{8.07}$	$5.65^{5.76}_{5.54}$	$5.07^{5.13}_{5.02}$	$2.33^{2.42}_{2.23}$
Window Size 50	$0.81^{0.85}_{0.77}$	$6.50^{6.59}_{6.40}$	$5.30^{5.42}_{5.18}$	$4.46^{4.53}_{4.39}$	$2.40^{2.52}_{2.30}$
Window Size $\sqrt{N_{n_0}}$	$0.91^{0.96}_{0.87}$	$5.57^{5.68}_{5.47}$	$4.82^{4.95}_{4.69}$	$3.90^{3.98}_{3.83}$	$2.94^{3.11}_{2.77}$
Initial Sequence: Chodera	$0.96^{1.00}_{0.92}$	$4.62^{4.74}_{4.51}$	$4.65^{4.83}_{4.48}$	$3.15^{3.24}_{3.06}$	$4.38^{4.60}_{4.15}$
Initial Sequence: Positive	$1.00^{1.04}_{0.95}$	$4.67^{4.78}_{4.55}$	$4.70^{4.89}_{4.52}$	$3.15^{3.24}_{3.06}$	$4.29^{4.51}_{4.06}$
Initial Sequence: Monotone	$0.99^{1.04}_{0.95}$	$4.66^{4.77}_{4.55}$	$4.63^{4.81}_{4.46}$	$3.18^{3.26}_{3.09}$	$4.32^{4.54}_{4.09}$
Initial Sequence: Convex	$0.98^{1.02}_{0.93}$	$4.68^{4.79}_{4.57}$	$4.63^{4.81}_{4.45}$	$3.22^{3.31}_{3.14}$	$4.32^{4.55}_{4.10}$
Initial Sequence: Smoothed Lag Convex	$0.98^{1.02}_{0.94}$	$4.67^{4.78}_{4.56}$	$4.65^{4.83}_{4.48}$	$3.23^{3.31}_{3.15}$	$4.29^{4.52}_{4.07}$

[a] All values in kcal mol^{-1}. Uncertainties are 95 % confidence intervals obtained by bootstrapping over 10000 iterations with replacement.

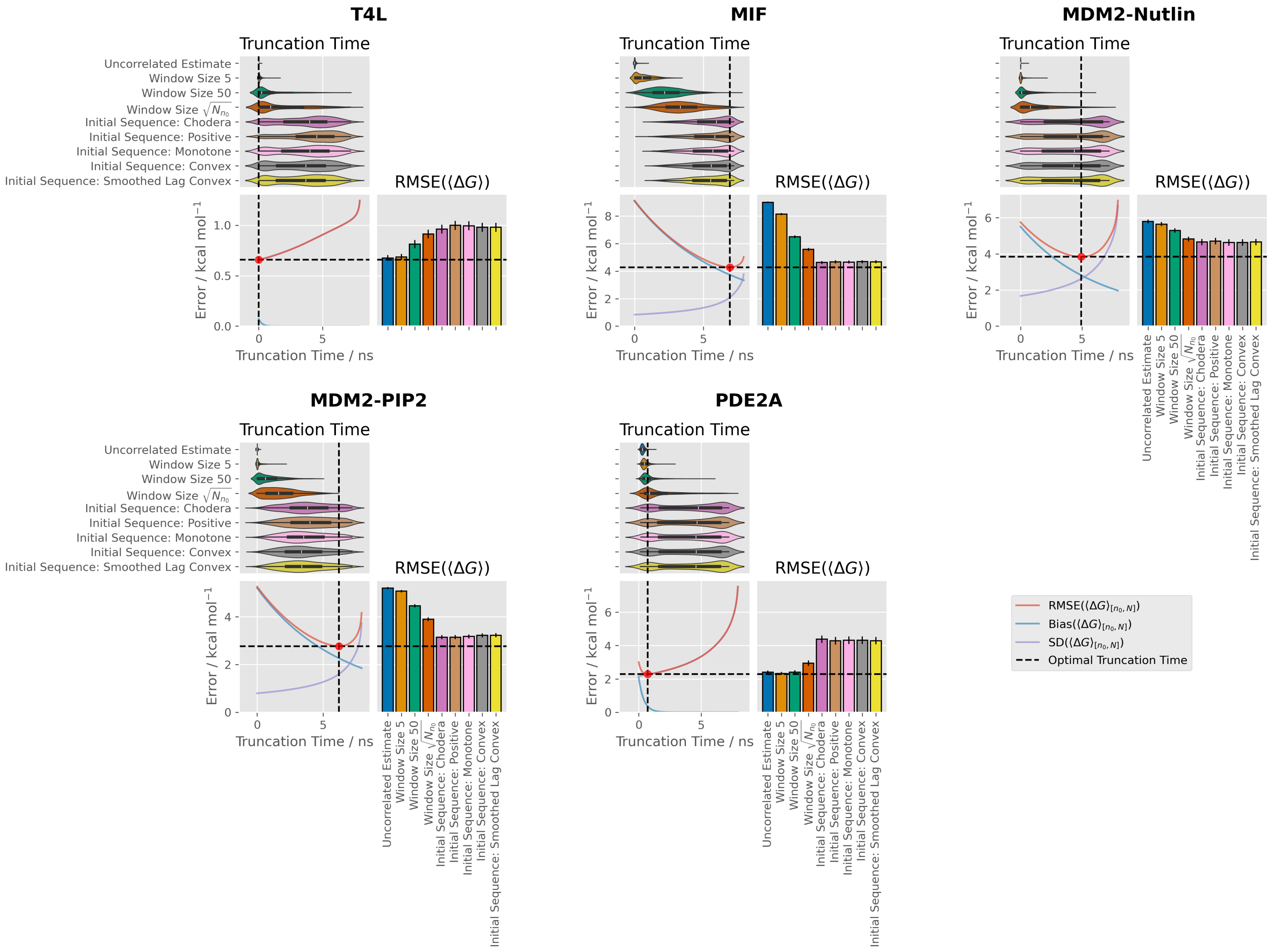

Figure S21: Discard times, RMSEs, and underlying time series properties for the bound vanish stage single λ window time series. The top panels show kernel density estimates of the distributions of times discarded with each method. The bottom left panels show the RMSEs which would be obtained with an infinitely large ensemble of synthetic time series with fixed truncation points. The red dot indicates the optimum fixed-time truncation point. Bottom right pannels show the RMSEs obtained over the full synthetic ensemble for each method. Uncertainties are 95 % confidence intervals which were obtained by 10000 iterations of boostrapping with replacement.

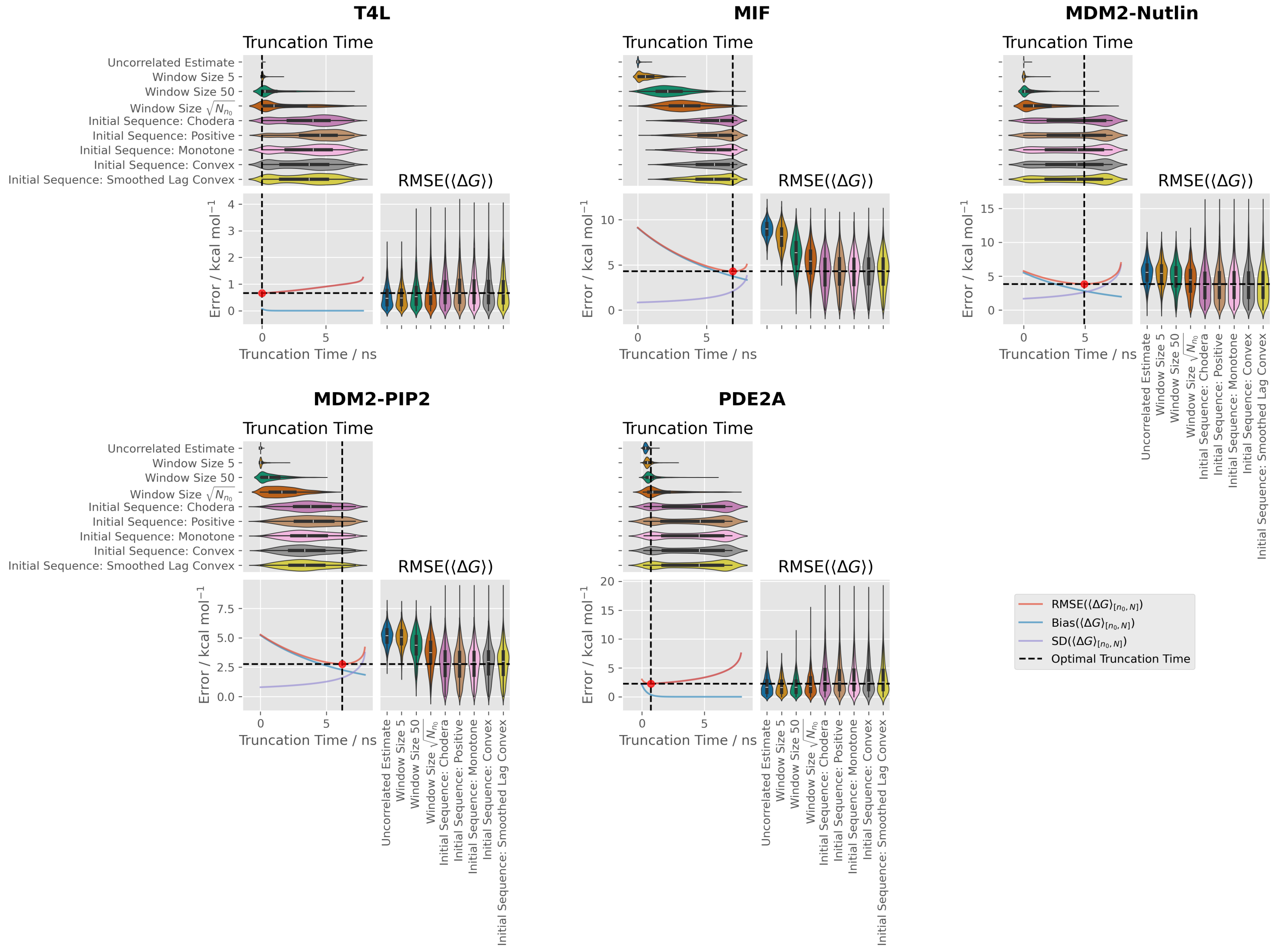

Figure S22: Discard times, unsigned errors, and underlying time series properties for the bound vanish stage single λ window time series. The top panels show kernel density estimates of the distributions of times discarded with each method. The bottom left panels show the RMSEs which would be obtained with an infinitely large ensemble of synthetic time series with fixed truncation points. The red dot indicates the optimum fixed-time truncation point. The bottom right pannels show the kernel density estimates of the distributions of unsigned errors obtained for each method.

S8 Coverage of Confidence Intervals for Truncated Free Vanish Data

Table S9: Coverage of 95% Confidence Intervals for Truncated Free Vanish Data[a]

**System**	**T4L**	**PDE2A**
**Dataset**	standard	standard
**Uncorrelated Estimate**	97	44
**Window Size 5**	97	50
**Window Size 50**	97	65
**Window Size** $\sqrt{N_{n_0}}$	97	71
**Initial Sequence: Chodera**	97	63
**Initial Sequence: Positive**	97	67
**Initial Sequence: Monotone**	97	64
**Initial Sequence: Convex**	97	63
**Initial Sequence: Smoothed Lag Convex**	97	63

[a] All coverages given as a % of ensemble members for which the 95% confidence interval included the true ensemble value (hence ideally all coverages should be $\approx$ 95%). Confidence intervals were calculated from each individual truncated time series using the same method used to select the truncation point by minimising the marginal standard error.

S9 The Variance Increase Resulting from Subsampling

A time series of length N is subsampled at intervals of S (so that the new series has length ($N_{\mathrm{Sub}} = \frac{N}{S}$). There is no initial bias and no truncation is performed. The variance of the mean of the subsampled time series, $\mathrm{Var}_{\mathrm{Trajs,Sub}}(\langle A\rangle_{[0,N_{\mathrm{Sub}}]})$, will be larger than that of the original time series, $\mathrm{Var}_{\mathrm{Trajs}}(\langle A\rangle_{[0,N]})$, by the factor

$$\frac{\mathrm{Var}_{\mathrm{Trajs,Sub}}(\langle A\rangle_{[0,N_{\mathrm{Sub}}]})}{\mathrm{Var}_{\mathrm{Trajs}}(\langle A\rangle_{[0,N]})} = \frac{\frac{1}{N_{\mathrm{Sub}}}\left(\gamma_0 + 2\sum_{t'=1}^{N_{\mathrm{Sub}}-1}\gamma'_{t'}\right)}{\frac{1}{N}\left(\gamma_0 + 2\sum_{t=1}^{N_{\mathrm{Sub}}-1}\gamma_t\right)} \tag{S8}$$

$$= S\frac{\gamma_0 + 2\sum_{t'=1}^{N_{\mathrm{Sub}}-1}\gamma'_{t'}}{\gamma_0 + 2\sum_{t=1}^{N-1}\gamma_t}, \tag{S9}$$

where $'$ denotes the lag times and autocovariances of the subsampled time series, and all other terms are defined in Section 2 of the main text. Taking out the common factor of γ_0, this can be rewritten as

$$\frac{\mathrm{Var}_{\mathrm{Trajs,Sub}}(\langle A\rangle_{[0,N_{\mathrm{Sub}}]})}{\mathrm{Var}_{\mathrm{Trajs}}(\langle A\rangle_{[0,N]})} = S\frac{1 + 2\sum_{t'=1}^{N_{\mathrm{Sub}}-1}\frac{\gamma'_{t'}}{\gamma_0}}{1 + 2\sum_{t=1}^{N-1}\frac{\gamma_t}{\gamma_0}} \tag{S10}$$

$$= S\frac{g_{\mathrm{Sub}}}{g}, \tag{S11}$$

where $g = 1 + 2\sum_{t=1}^{N-1}\frac{\gamma_t}{\gamma_0}$ is the statistical inefficiency of the original time series, and g_{Sub} is the statistical inefficiency of the subsampled time series. When there is no autocorrelation, $g = g_{\mathrm{Sub}}$ and the increase in variance is proportional to the subsampling interval. This is the worst case scenario. When the time series is autocorrelated, subsampling reduces the autocorrelation, meaning that $g > g_{\mathrm{Sub}}$ and $\frac{g_{\mathrm{Sub}}}{g} < 1$, hence proportional increase in error is less than if the samples were uncorrelated (when S is constant).

Often, data are subsampled according to their statistical inefficiency.[4] Assuming a perfect estimate of the statistical inefficiency, $S = g$ and

$$\frac{\mathrm{Var}_{\mathrm{Trajs,Sub}}(\langle A\rangle_{[0,N_{\mathrm{Sub}}]})}{\mathrm{Var}_{\mathrm{Trajs}}(\langle A\rangle_{[0,N]})} = g_{\mathrm{Sub}}. \tag{S12}$$

Following Janke,[5] we explore the idealised case when an autocorrelation function is a single exponential with decay constant τ. Then, the statistical inefficiency is

$$g = 1 + 2\sum_{t=1}^{N-1}\frac{\gamma_t}{\gamma_0} \tag{S13}$$

$$= 1 + 2\sum_{t=1}^{N-1}e^{-\frac{t}{\tau}}. \tag{S14}$$

Assuming that $N \gg \tau$,

$$g \approx 1 + 2\sum_{t=1}^{\infty}e^{-\frac{t}{\tau}} = -1 + 2\sum_{t=0}^{\infty}e^{-\frac{1}{\tau}} \tag{S15}$$

$$= -1 + \frac{2}{1 - e^{-\frac{1}{\tau}}} \tag{S16}$$

$$= \frac{1 + e^{-\frac{1}{\tau}}}{1 - e^{-\frac{1}{\tau}}} \tag{S17}$$

$$= \coth\left(\frac{1}{2\tau}\right) \tag{S18}$$

The subsampled decay constant, τ_{Sub}, is related to τ by

$$e^{-\frac{t'}{\tau_{\mathrm{Sub}}}} = e^{-\frac{t}{S\tau_{\mathrm{Sub}}}} = e^{-\frac{t}{\tau}}, \tag{S19}$$

hence

$$\tau_{\text{Sub}} = \frac{\tau}{S}. \tag{S20}$$

When subsampling is performed at the interval of the perfectly-estimated statistical inefficiency, then

$$\tau_{\text{Sub}} = \frac{\tau}{\coth\left(\frac{1}{2\tau}\right)} \tag{S21}$$

$$= \tau \tanh\left(\frac{1}{2\tau}\right). \tag{S22}$$

If $\tau \gg \frac{1}{2}$, then $\frac{1}{2\tau} \ll 1$ and

$$\tau_{\text{Sub}} \approx \frac{\tau}{2\tau} = \frac{1}{2}. \tag{S23}$$

Then

$$\frac{\text{Var}_{\text{Trajs,Sub}}(\langle A \rangle_{[0,N_{\text{Sub}}]})}{\text{Var}_{\text{Trajs}}(\langle A \rangle_{[0,N]})} \approx \coth(1) = 1.31. \tag{S24}$$

Hence, for a stationary time series with a single exponential autocorrelation function, when the decay constant is much greater than $\frac{1}{2}$ (e.g. the half-life is much greater than the sampling interval) and the number of samples is much greater than the decay constant, subsampling increases the variance of the mean by 31%.

References

(1) Chodera, J. D. A Simple Method for Automated Equilibration Detection in Molecular Simulations. *J. Chem. Theory Comput.* **2016**, *12*, 1799–1805.

(2) Chodera, J. D.; Swope, W. C.; Pitera, J. W.; Seok, C.; Dill, K. A. Use of the Weighted Histogram Analysis Method for the Analysis of Simulated and Parallel Tempering Simulations. *J. Chem. Theory Comput.* **2007**, *3*, 26–41.

(3) Shirts, M. R.; Chodera, J. D. Statistically Optimal Analysis of Samples from Multiple Equilibrium States. *J. Chem. Phys.* **2008**, *129*, 124105.

(4) Hahn, D.; Bayly, C.; Boby, M. L.; Macdonald, H. B.; Chodera, J.; Gapsys, V.; Mey, A.; Mobley, D.; Benito, L. P.; Schindler, C.; Tresadern, G.; Warren, G. Best Practices for Constructing, Preparing, and Evaluating Protein-Ligand Binding Affinity Benchmarks [Article v1.0]. *Living J. Comput. Mol. Sci.* **2022**, *4*, 1497–1497.

(5) Janke, W. In *Quantum Simulations of Complex Many-Body Systems: From Theory to Algorithms*; Grotendorst, J., Marx, D., Murmatsu, A., Eds.; John von Neumann Institute for Computing, 2002; Vol. 10.
